# Gfi1aa/Lsd1 Facilitates Hemangioblast Differentiation Into Primitive Erythrocytes by Targeting *etv2* and *sox7* in Zebrafish

**DOI:** 10.3389/fcell.2021.786426

**Published:** 2022-01-12

**Authors:** Mei Wu, Qi Chen, Jing Li, Yue Xu, Junwei Lian, Yongxiang Liu, Ping Meng, Yiyue Zhang

**Affiliations:** ^1^ Division of Cell, Developmental and Integrative Biology, School of Medicine, South China University of Technology, Guangzhou, China; ^2^ Department of Developmental Biology, School of Basic Medical Sciences, Southern Medical University, Guangzhou, China; ^3^ Shenzhen Key Laboratory for Neuronal Structural Biology, Biomedical Research Institute, Shenzhen Peking University-The Hong Kong University of Science and Technology Medical Center, Shenzhen, China

**Keywords:** zebrafish, hemangioblast differentiation, primitive erythrocyte, Gfi1aa, *etv2*, *sox7*

## Abstract

The first wave of hematopoiesis is the primitive hematopoiesis, which produces embryonic erythroid and myeloid cells. Primitive erythrocytes are thought to be generated from bipotent hemangioblasts, but the molecular basis remains unclear. Transcriptional repressors Gfi1aa and Gfi1b have been shown to cooperatively promote primitive erythrocytes differentiation from hemangioblasts in zebrafish. However, the mechanism of these repressors during the primitive wave is largely unknown. Herein, by functional analysis of zebrafish *gfi1aa* ^*smu10*^, *gfi1b* ^*smu11*^, *gfi1ab* ^*smu12*^ single, double, and triple mutants, we found that Gfi1aa not only plays a predominant role in primitive erythropoiesis but also synergizes with Gfi1ab. To screen Gfi1aa downstream targets, we performed RNA-seq and ChIP-seq analysis and found two endothelial transcription factors, *etv2* and *sox7*, to be repressed by Gfi1aa. Genetic analysis demonstrated Gfi1aa to promote hemangioblast differentiation into primitive erythrocytes by inhibiting both *etv2* and *sox7* in an Lsd1-dependent manner. Moreover, the H3K4me1 level of *etv2* and *sox7* were increased in *gfi1aa* mutant. Taken together, these results suggest that Gfi1aa/Lsd1-dependent *etv2/sox7* downregulation is critical for hemangioblast differentiation during primitive hematopoiesis by inhibition of endothelial specification. The different and redundant roles for Gfi1(s), as well as their genetic and epigenetic regulation during primitive hematopoiesis, help us to better know the molecular basis of the primitive hematopoiesis and sheds light on the understanding the Gfi1(s) related pathogenesis.

## Introduction

Hematopoiesis in vertebrates includes two distinct waves, the primitive wave and the definitive wave. In the primitive wave of mammals, both primitive erythroid and endothelial cells originate from the mesoderm and then aggregate and form the yolk sac blood island (Baron et al., 2012; (Garcia and Larina, 2014). In zebrafish, primitive erythroblasts originate from the lateral plate mesoderm (LPM) and then migrate to the intermediate cell mass, which is equivalent to the yolk sac blood island in mammals (Chen and Zon, 2009). Angioblasts (endothelial precursor cells) migrate to the midline from the LPM and form the vascular cord (Jin et al., 2005). Both hematopoietic and endothelial cells are thought to be derived from a common progenitor known as the hemangioblast (Lancrin et al., 2009; (Lacaud and Kouskoff, 2017), which was first proposed by Muttay in the early chick embryo (Murray, 1932). Although hemangioblasts have not been detected in mice (likely due to rare numbers), in zebrafish, a labeled gastrula-stage cell was shown to generate both hematopoietic and endothelial cells (Vogeli et al., 2006). This result suggests that the zebrafish is a model organism by which to define hemangioblast differentiation.

A series of transcription factors (e.g., *Scl/Tal1* (Gering et al., 1998), *Lmo2* (Patterson et al., 2007), *Gata2* (Lugus et al., 2007), *Etv2* (Liu and Patient, 2008), and *Fli1* (Hart et al., 2000; (Spyropoulos et al., 2000; (Liu et al., 2008)) have been found that are expressed in both hematopoietic and endothelial cells. Genetic mutation of these transcription factors results in both hematopoiesis and vasculogenesis dysfunction (Gering et al., 1998; (Hart et al., 2000; (Spyropoulos et al., 2000; (Lugus et al., 2007; (Patterson et al., 2007; (Liu and Patient, 2008; (Liu et al., 2008), which provides molecular evidence for the existence of a common hemangioblast. Yet, the progression and regulation of hemangioblast differentiation, especially the molecular pathways by which hemangioblast transition to endothelial and hematopoietic cells, are largely unknown.

Gfi1 family members are reported to be involved in hemangioblast differentiation (Moore et al., 2018). Zebrafish has three Gfi1(s) paralogs: Gfi1aa and Gfi1ab are thought to be orthologs of mammalian GFI1 (Wei et al., 2008; (Cooney et al., 2013), and Gfi1b is considered to be the mammalian GFI1B’s ortholog (Cooney et al., 2013). It is reported that Gfi1aa promotes primitive erythropoiesis (Wei et al., 2008), subsequently, Gfi1b is shown synergistically with Gfi1aa to promote primitive erythroblast differentiation from hemangioblasts (Moore et al., 2018), but the molecular basis for their function is largely unclear. Gfi1ab is not expressed in primitive hematopoietic regions (Dufourcq et al., 2004), but its expression is increased in the absence of Gfi1aa (Thambyrajah et al., 2016b), suggesting the unclear role of Gfi1ab in primitive hematopoiesis. In addition, the histone demethylase, Lsd1, which demethylates mono- and di-methylated H3K4, is a co-factor of Gfi1 (Saleque et al., 2007) and critical for Gfi1aa transcription repression (Velinder et al., 2016), and its deficiency blocks primitive erythropoiesis (Takeuchi et al., 2015). Our previous study also has shown Gfi1aa inhibited *cebpa* expression to control neutrophil progenitor expansion was dependent upon Lsd1 (Wu et al., 2021). However, whether Gfi1aa regulates hemangioblast differentiation is dependent upon Lsd1 remains unknown. As such, the different and redundant roles for Gfi1(s), as well as their genetic and epigenetic regulation during primitive erythrocytes differentiated from hemangioblast, are not fully understood.

In this study, we assessed the role of the three zebrafish Gfi1 orthologs during primitive hematopoiesis and found that Gfi1aa, rather than Gfi1b and Gfi1ab, played a predominant role in hemangioblast differentiation to primitive erythroid cells. We screened potential Gfi1aa downstream targets by performing RNA-seq and ChIP-seq analysis and then verified genetic regulation. We found that Gfi1aa, with the help of histone demethylase Lsd1, downregulates *etv2* and *sox7*, suppressing hemangioblast endothelial potential and promoting erythroid differentiation.

## Materials and Methods

### Zebrafish Husbandry

Zebrafish were raised and maintained as described (Westerfield, 2000). The following strains were used: the AB strain, the *gfi1aa* ^*smu10*^ mutant (Wu et al., 2021), the *gfi1b* ^*smu11*^ mutant, and the *gfi1ab* ^*smu12*^ mutant. All zebrafish studies were approved by the South China University of Technology Animal Advisory Committee.

### Generation *gfi1b* and *gfi1ab* Mutants

For the *gfi1b* ^*smu11*^ mutant and the *gfi1ab* ^*smu12*^ mutant, the gRNA (*gfi1b*: 5′- gga​gga​aac​tct​gcc​agc​tg-3′, *gfi1ab*: 5′- ggt​act​cgg​ggt​gtg​aaa​tc-3′) was co-injected with Cas9 protein (NEB, MA, United States; M0646M) into one-cell stage embryos, the gRNAs were synthesized as described (Chang et al., 2013). The raising and screening of mutants were performed as previously described (Chang et al., 2013; (Liu et al., 2014). The genotyping primers were listed in Supplementary Table S1.

### Whole Mount *in situ* Hybridization (WISH) and Immunofluorescence

Probes synthesis and WISH were carried out as described (Thisse and Thisse, 2008). The following probes were synthesized: *gata1*, *alas2*, *scl*, *gata2a*, *fli1*, *etv2*, *sox7*, and *flk1*. Embryos for immunofluorescence were fixed with 4% paraformaldehyde at 23 hpf and dehydrated by methanol. Then the embryos were permeabilized by acetone and stained with GFP antibody (Abcam, Cambridge, UK; ab6658).

### Transgenic Zebrafish Generation and Heat Shock Treatment

For Tg (*hsp70:gfi1aa-eGFP*) transgenic zebrafish, the embryos injected with *pTol-hsp70-eGFP* construct and transposase mRNA (Wu et al., 2021) were raised to adult, then the stable transgenic lines were screened as previously described (Westerfield, 2000). To overexpress *gfi1aa*, 12 hpf embryos were heat shocked for 2 h at 39°C, then the GFP + embryos were picked out for subsequent experiments.

### RNA Isolation and RNA-Seq

The *gfi1aa* ^*smu10*^ mutant, *gfi1b* ^*smu11*^ mutant, and *gfi1ab* ^*smu12*^ mutant were generated from *gfi1aa* ^*smu10/+*^, *gfi1b* ^*smu11/+*^, and *gfi1ab* ^*smu12/+*^ intercrossed embryos by genotyping respectively. The *gfi1aa* ^*smu10*^ *gfi1b* ^*smu11*^ mutant, *gfi1aa* ^*smu10*^ *gfi1ab* ^*smu12*^ mutant, and the *gfi1aa* ^*smu10*^ *gfi1b* ^*smu11*^ *gfi1ab* ^*smu12*^ mutant were generated from *gfi1aa* ^*smu10/+*^ *gfi1b* ^*smu11*^, *gfi1aa* ^*smu10/+*^ *gfi1ab* ^*smu12*^, and *gfi1aa* ^*smu10*^ *gfi1b* ^*smu11*^ *gfi1ab* ^*smu12/+*^ intercrossed embryos by genotyping respectively. Then, RNA from *gfi1*-related single, double, and triple mutants as well as WT (wild type siblings) embryos was extracted with TRIzol reagent (Invitrogen, CA, United States; 15596026). Sequencing libraries were generated using the NEBNext® UltraTM RNA Library Prep Kit for Illumina® RNA (NEB; E7770) according to the manufacturer’s instructions.

### Bioinformatic Analysis

For RNA-seq data, the sequencing reads were mapped to Ensemble zebrafish reference genome (GRCz11) using STAR alignment software (Dobin et al., 2013). The differential gene expression analysis was performed by DESeq2 (Love et al., 2014). For GO enrichment analysis, the Metascape website (https://metascape.org/gp) (Zhou et al., 2019) was used.

### Chromatin Immunoprecipitation-Polymerase Chain Reaction (ChIP-PCR)

Gfi1aa-GFP ChIP assay was performed as previously described (Wu et al., 2021). In detail, ∼250 WT embryos injected with the *hsp-gfi1aa-eGFP* plasmid or *hsp-eGFP* plasmid were heat-shocked and collected at 15 hpf, then the samples were performed by cross-linking, sonication, antibody binding, washing, reverse-cross linking, and ChIP DNA extraction. The ChIP DNA was assessed by qPCR with a LightCycler 96 system (Roche). The comparable WT group and *gfi1aa^smu10^* mutant group were respectively intercrossed for H3K4me1 ChIP. About 200 embryos of each group were collected at 15 hpf and ChIP DNA was extracted as above. The *etv2* ChIP-qPCR primers are used as previously described (Takeuchi et al., 2015), and *sox7* ChIP-qPCR primer is listed in Supplementary Table S1.

### *In vivo* Transient GFP Reporter Assay

For the transient GFP reporter assay, pTol-*etv2*-eGFP and pTol-*sox7*-eGFP plasmids were constructed for GFP expression under the control of *etv2* or *sox7* regulatory regions. For the pTol-*etv2*-eGFP plasmid, the 3.4 kb *etv2* promoter (Veldman and Lin, 2012), containing *etv2 up-1* to *intron-2* region, was cloned by PCR (Primers are listed in Supplementary Table S1) from genomic DNA and inserted into the pTol vector to drive GFP. For the pTol-*sox7*-eGFP plasmid, the 0.7 kb promoter (containing the Gfi1aa binding peak) was cloned and constructed as above. Then, 100 ng/μL of the construct was injected into the WT control and *gfi1aa^smu10^* mutant embryos.

### Microinjection of Morpholinos (MOs)

MOs for *etv2* (5′-cac​tga​gtc​ctt​att​tca​cta​tat​c-3′) (Sumanas and Lin, 2006), *lsd1* (5′-gtt​att​cac​acc​ttg​ttg​aga​ttt​c-3′) (Takeuchi et al., 2015), and *sox7* (5′-acg​cac​tta​tca​gag​ccg​cca​tgt​g-3′) (Cermenati et al., 2008) were synthesized by Gene Tools and dissolved in water. One-cell stage embryos were collected and injected. For double knockdown, the final concentration of 0.005 pmol *etv2* MO and 0.5 pmol *sox7* MO were used.

### Statistical Analysis

GraphPad Prism 7.0 was used for analysis of experimental data. The Fisher’s exact test was used to compare the difference between two categorical variables. The Unpaired *t-*test was used to compare the mean difference of two independent groups. The *p*-value less than 0.05 was considered statistically significant.

## Results

### Gfi1ab Synergizes With Gfi1aa to Promote Primitive Erythropoiesis

**FIGURE 1:**
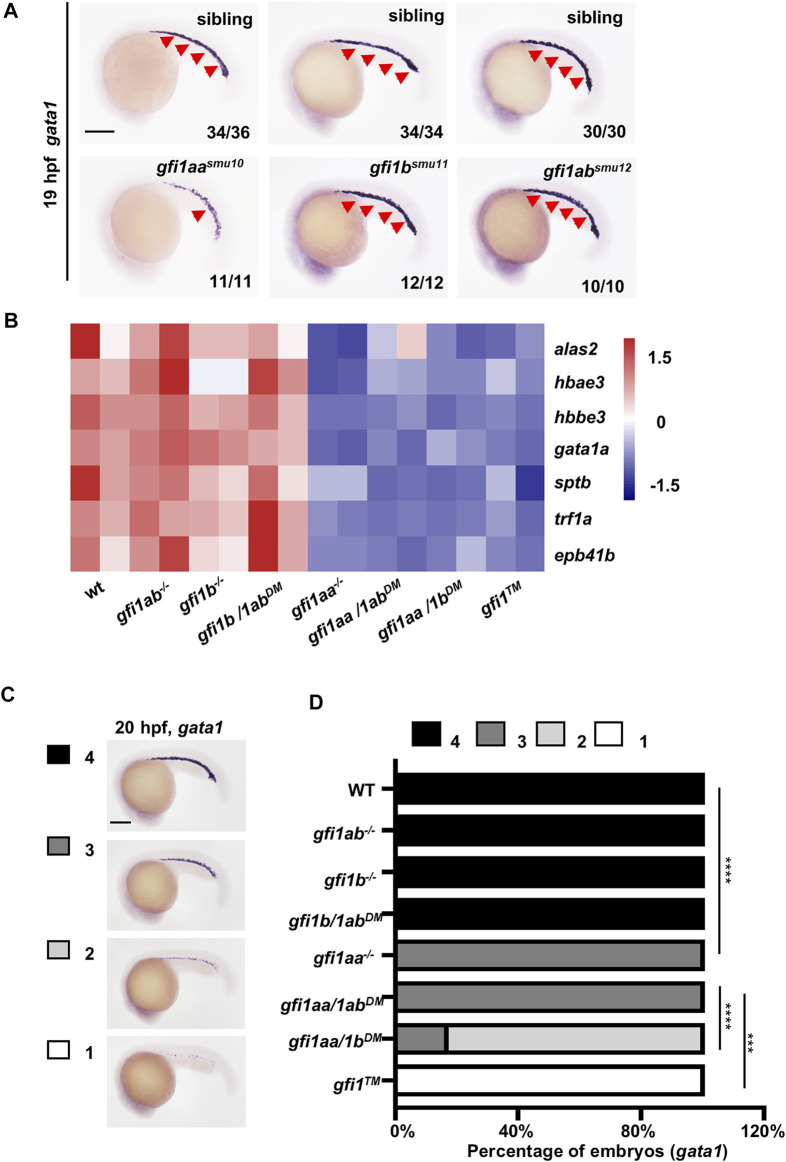
*gfi1aa* plays the key role in primitive erythropoiesis **(A)** Expression of *gata1* was increased in *gfi1aa* ^*smu10*^ mutants compared to siblings, whereas *gfi1b* ^*smu11*^ and *gfi1ab* ^*smu12*^ mutants show normal *gata1* expression at 19 hpf by WISH. The numbers in the lower right corner indicate representative expression embryo numbers of the indicated marker. Scale bar: 200 μm **(B)** Heatmap of WT, *gfi1aa* ^*−/−*^, *gfi1b* ^*−/−*^, *gfi1ab* ^*−/−*^ signal mutant, *gfi1aa/1b* ^*DM*^, *gfi1aa/1ab* ^*DM*^, *gfi1b/1ab* ^*DM*^ double mutant and *gfi1* ^*TM*^ triple mutant shows the gene expression levels of erythroid genes (*alas2*, *hbae3*, *hbbe3*, *gata1a*, *sptb*, *trf1a,* and *epb41b*). The color scale indicated the expression level **(C,D)** Expression of *gata1* was decreased in *gfi1aa* related mutant (*gfi1aa* ^*−/−*^, *gfi1aa/1ab* ^*DM*^, *gfi1aa/1b* ^*DM*^, and *gfi1* ^*TM*^) compared to WT and other mutants at 20 hpf by WISH **(C)** The *gfi1aa* ^*+/-*^; *gfi1b* ^*+/-*^; *gfi1ab* ^*+/-*^ intercross embryos were divided into four categories according to *gata1* expression **(D)** The percentage of WT, *gfi1aa* ^*−/−*^, *gfi1b* ^*−/−*^, *gfi1ab* ^*−/−*^ signal mutant, *gfi1aa/1b* ^*DM*^, *gfi1aa/1ab* ^*DM*^, *gfi1b/1ab* ^*DM*^ double mutant and *gfi1* ^*TM*^ triple mutant according to the categories (****p < 0.0001, ***p < 0.001, Fisher exact tests, n ≥ 10 for each group).

To determine the relationship of three Gfi1(s) to primitive hematopoiesis, we utilized a *gfi1aa* ^*smu10*^ zebrafish mutant (Wu et al., 2021)) and generated *gfi1b* ^*smu11*^ and *gfi1ab* ^*smu12*^ zebrafish mutants with CRISPR/Cas9 technology (Supplementary Figure one). Similar to the *gfi1aa* ^*smu10*^ mutant (Wu et al., 2021), *gfi1b* ^*smu11*^ and *gfi1ab* ^*smu12*^ mutants, with a 58-nt insertion (Supplementary Figure S1A) and a 1-nt deletion (Supplementary Figure S1B), respectively, were predicted to disrupt C2H2 type zinc finger domains. To identify the respective roles of Gfi1 members in primitive erythropoiesis, we compared erythroid marker, *gata1*, expression by WISH in each mutant. We found the expression of *gata1* was decreased in *gfi1aa* ^*smu10*^ mutant embryos compared to their siblings, while no apparent difference in the *gfi1b* ^*smu11*^ mutant was found compared to siblings (Figure 1A), which is consistent with previously described *gfi1aa* ^*qmc551*^ and *gfi1b* ^*qmc554*^ mutants (Moore et al., 2018). We also monitored the phenotype of *gfi1ab* ^*smu12*^ mutants and found *gata1* expression was no altered (Figure 1A), suggesting that loss of *gfi1ab* does not affect primitive erythropoiesis.

To further identify the relationships among the three *gfi1* members, we performed RNA-seq on wild-type (WT), *gfi1aa* ^*smu10*^, *gfi1b* ^*smu11*^, *gfi1ab* ^*smu12*^ single mutant, *gfi1aa* ^*smu10*^ *gfi1b* ^*smu11*^, *gfi1aa* ^*smu10*^ *gfi1ab* ^*smu12*^, *gfi1b* ^*smu11*^ *gfi1ab* ^*smu12*^ double mutant and *gfi1aa* ^*smu10*^ *gfi1b* ^*smu11*^ *gfi1ab* ^*smu12*^ triple mutant (hereafter referred to as *gfi1aa* ^*−/−*^, *gfi1b* ^*−/−*^, *gfi1ab* ^*−/−*^, *gfi1aa/1b* ^*DM*^, *gfi1aa/1ab* ^*DM*^, *gfi1b/1ab* ^*DM*^, and *gfi1* ^*TM*^). As shown in the RNA-seq heatmap, we found that erythroid markers (*alas2*, *hbae3*, *hbbe3*, *gata1a*, *sptb*, *trf1a,* and *epb41b*) were decreased in *gfi1aa* related mutants (*gfi1aa* ^*−/−*^, *gfi1aa/1b* ^*DM*^, *gfi1aa/1ab* ^*DM*^ and *gfi1* ^*TM*^) compared to WT and *gfi1aa* unrelated mutants (*gfi1b* ^*−/−*^, *gfi1ab* ^*−/−*^ and *gfi1b/1ab* ^*DM*^) (Figure 1B). For validation, we further performed *gata1* WISH on these mutants. Consistent with the RNA-seq data, the expression of *gata1* was not altered in WT and *gfi1aa* unrelated mutants (Figures 1C,D). The expression of *gata1* was decreased in *gfi1aa* mutants and *gfi1aa/1ab* ^*DM*^, further decreased in *gfi1aa/1b* ^*DM*^ and the most decreased in *gfi1* ^*TM*^ (Figures 1C,D). We then explored the genetic interplay among *gfi1s* and found *gfi1b* was decreased in *gfi1aa*-related mutants whereas *gfi1ab* was ectopic increased in *gfi1aa*-related mutants (Supplementary Figure S2A,B), suggesting *gfi1aa* dominates the expression of *gfi1b* and *gfi1ab*. These data indicate that Gfi1aa plays a predominant role in promoting primitive erythropoiesis, and that Gfi1ab, together with Gfi1b, play synergistic roles in the process.

### Identification of Gfi1aa Target Genes That Promote Hemangioblast Differentiation Into Primitive Erythroid Cells

**FIGURE 2:**
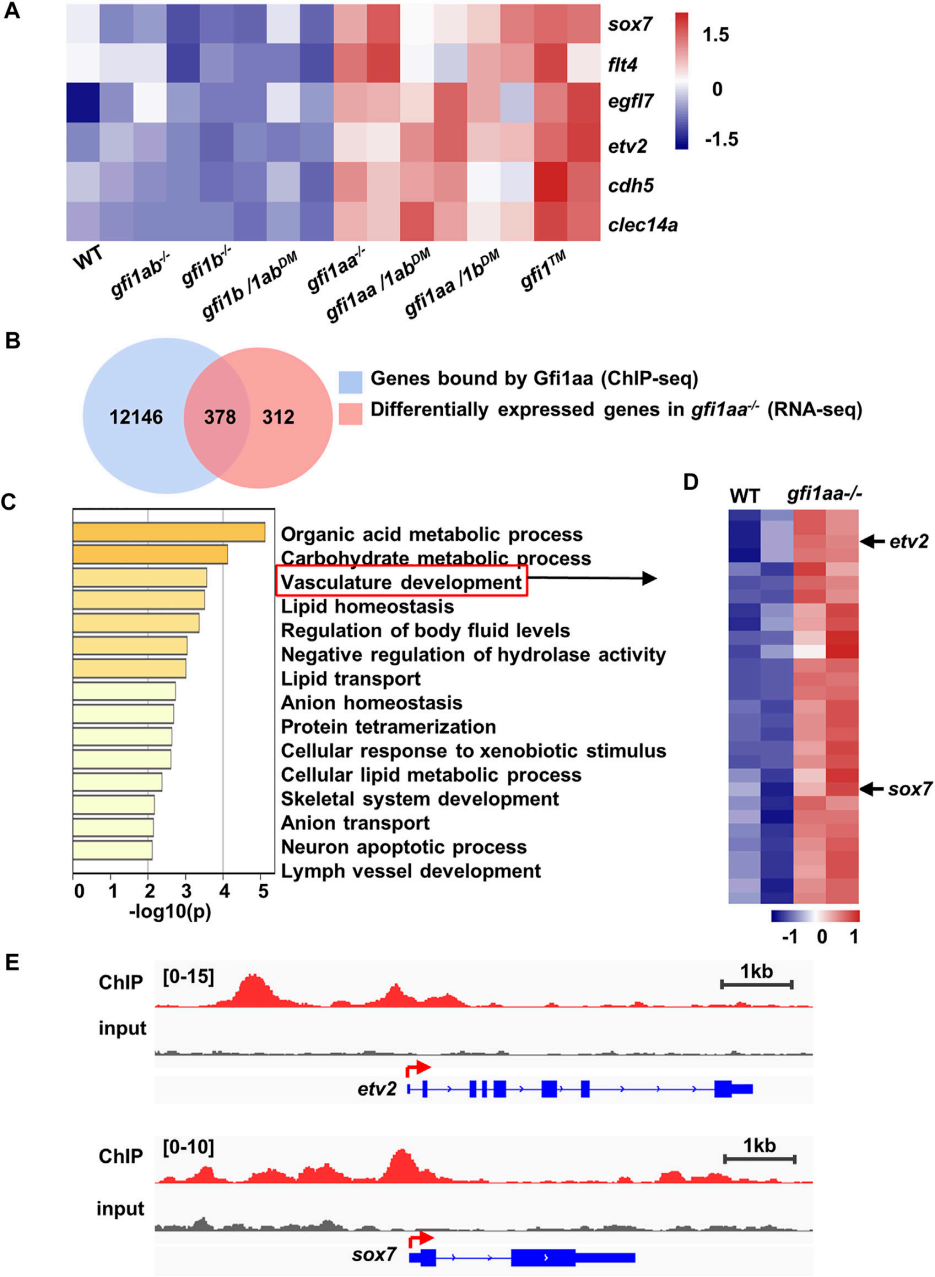
Gfi1aa could bind to *etv2* and *sox7* regulator regions **(A)** Endothelial genes were increased in *gfi1aa* related mutants. Heatmap of WT, *gfi1aa* ^*−/−*^, *gfi1b* ^*−/−*^, *gfi1ab* ^*−/−*^ signal mutant, *gfi1aa/1b* ^*DM*^, *gfi1aa/1ab* ^*DM*^, *gfi1b/1ab* ^*DM*^ double mutant and *gfi1* ^*TM*^ triple mutant showed the gene expression levels of endothelial genes (*sox7*, *flt4*, *cdh5*, *clec14a*, *etv2,* and *egfl7*). The color scale indicated the expression level **(B)** Combinational analysis of *gfi1aa* ^*−/−*^ RNA-seq and Gfi1aa-eGFP ChIP-seq. 378 genes were overlapped between 690 up-regulated genes in *gfi1aa* ^*−/−*^ mutant and 12,524 genes bound by Gfi1aa **(C)** Go enrichment analysis of the 378 combinational genes. Vasculature development GO term was indicated by the red box **(D)** Heat map of WT and *gfi1aa* ^*−/−*^ mutant showed the vasculature development genes expression levels from **(C)**. The color scale indicated the expression level **(E)** Visualization of Gfi1aa binding sites on *etv2* **(top)** and *sox7* **(bottom)** indicated by Gfi1aa ChIP-seq (red) compared to input control (grey) through integrative genomics viewer (IGV).

Gfi1aa and Gfi1b control primitive erythroblast differentiation by inhibition of endothelial programs (Moore et al., 2018), but the regulatory mechanisms and the key downstream factors are largely unknown. We speculated that Gfi1aa target genes probably exist in the upregulated genes of *gfi1aa* ^*−/−*^ mutant RNA-seq. Through Gene Ontology (GO) enrichment analysis of upregulated genes, we found vasculature development to be the most enriched GO term (Supplementary Figure 3A). Representative endothelial markers (including *sox7*, *flt4*, *cdh5*, *clec14a*, *etv2,* and *egfl7* (Kaipainen et al., 1995; (Parker et al., 2004; (Sumanas et al., 2005; (Pham et al., 2007; (Cermenati et al., 2008)) were all upregulated in *gfi1aa* ^*−/−*^ mutant RNA-seq (Supplementary Figure S3B). By comparison of the differential expression of the endothelial markers among all *gfi1* mutants, we found representative genes were specifically upregulated in all *gfi1aa*-related mutants (Figure 2A), and particularly upregulated in *gfi1* ^*TM*^. These data suggest that Gfi1aa, rather than Gfi1b or Gfi1ab, plays a predominant role in the inhibition of endothelial programs during hemangioblast differentiation into primitive erythrocytes.

As Gfi1(s) function as transcription repressors, it is important to know which genes are directly targeted by Gfi1(s). By reanalyzing our previously performed Gfi1aa-eGFP ChIP-seq data (Wu et al., 2021), we found 12,524 genes bound by Gfi1aa with analyzing the peaks located 2 kb upstream and 2 kb downstream from the transcription start site (TSS) (Figure 2B). When RNA-seq upregulated genes of the *gfi1aa* ^*−/−*^ mutant were combined with the Gfi1aa ChIP targeted genes, we identified 378 candidates that may be directly targeted and transcriptionally suppressed by Gfi1aa (Figure 2B). As expected, the GO term analysis for the 378 candidate targets showed that the vasculature development pathway was highly enriched (Figure 2C). 29 endothelial associated genes were found to be involved in the pathway (Figure 2D). We then compared the differential expression of these genes among all *gfi1* mutants and found *sox7*, *flt4*, *egfl7*, *cdh5*, *etv2* were upregulated in *gfi1aa*-related mutants (Supplementary Figure S4A).

As transcription factors are thought to be critical for cell fate determination, we speculated that some transcription factors may be responsible for Gfi1aa involvement in primitive erythropoiesis. *Etv2* and *Sox7*, two hemangioblast markers, were both highly expressed in mesodermal precursors but downregulated in differentiated hematopoietic cells (Gandillet et al., 2009; (Costa et al., 2012; (Veldman and Lin, 2012; (Sumanas and Choi, 2016). Previous studies showed that overexpression of either one promoted endothelial specification (Kataoka et al., 2011; (Costa et al., 2012). Moreover, *etv2* and *sox7* genes were highly bound by Gfi1aa-eGFP and their mRNAs were upregulated in *gfi1aa*-related mutants (Figures 2D,E, Supplementary Figure S4A). Therefore, we speculate that Gfi1aa may directly target and suppress *etv2* and *sox7* to promote hemangioblast differentiation into primitive erythrocytes by preventing the endothelial specification program.

### Gfi1aa Directly Targets *etv2* and *sox7* and Suppresses Their Transcription

**FIGURE 3:**
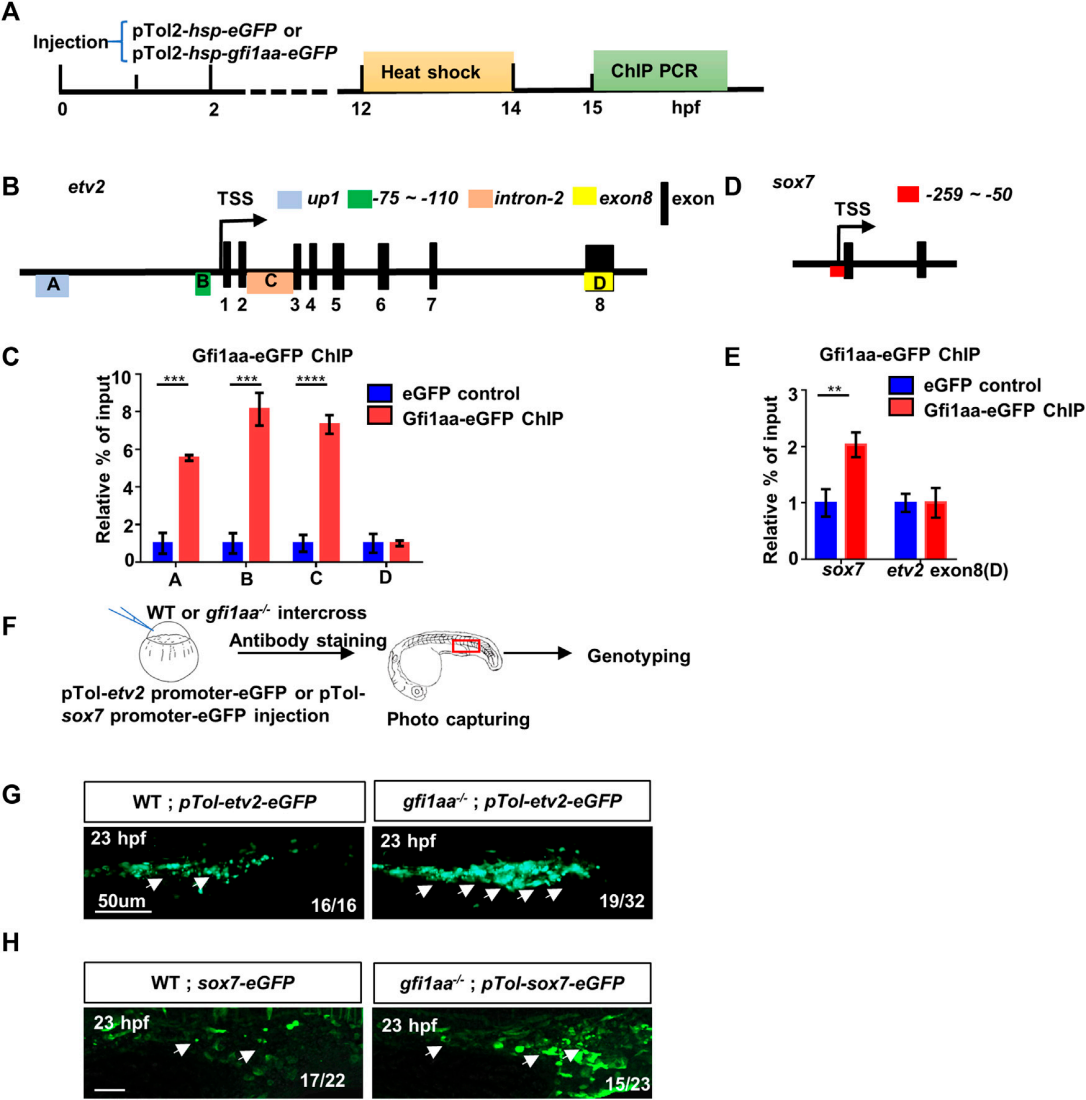
Gfi1aa directly represses *etv2* and *sox7* expression **(A)** Workflow of Gfi1aa-eGFP ChIP-PCR assay **(B)** Schematic diagram of *etv2* gene structure. Three regulator regions *up1* (Box A, blue colored), *-75 ∼ -110 bp* (Box B, green colored), *intron-2* (Box C, orange colored) were showed on the gene structure, black boxes indicated the exons, *exon-8* (Box D, yellow colored) as the control region. Box-(A–D) represented the detected region for *etv2* ChIP PCR products **(C)** ChIP-qPCR showed Gfi1aa enriched in *etv2* regulatory regions compared to eGFP control (*up1*, 5.5-fold; *-75 ∼ -110bp*, 8.1-fold; *intron-2*, 7.3-fold), the results were mean ± SD and generated from three independent experiments (****p < 0.0001, ***p < 0.001, *t*-test) **(D)** Schematic diagram of *sox7* gene structure. Red box represented the detected region for *sox7* ChIP PCR products **(E)** ChIP-qPCR showed 2-fold of Gfi1aa enriched in *sox7* regulatory regions compared to eGFP control. The results were mean ± SD and generated from three independent experiments (**p < 0.01, *t*-test) **(F–H)** Gfi1aa was a transcription repressor for *etv2* and *sox7* **(F)** The scheme of transient GFP reporter assay for *pTol-etv2-eGFP* construct and *pTol-sox7-eGFP* construct. The red box indicated the image region **(G,H)** Transient expression of *pTol-etv2-eGFP* construct **(G)** and *pTol-sox7-eGFP* construct **(H)** in WT and *gfi1aa* ^*−/−*^ mutant embryos. Fluorescence in the ICM region was monitored at 23 hpf. Scale bar: 50 μm.

To test the hypothesis, we first validated our digital data. For validation of ChIP-seq results, we performed a ChIP-PCR assay using the pTol2-*hsp-gfi1aa-eGFP* construct to assess whether Gfi1aa could bind to *etv2* and *sox7* regulatory regions (Figure 3A). Previous data showed that three *etv2* regulator regions (*up1*, *-110 ∼ -35bp* and *intron-2*) recapitulated *etv2* expression (Veldman and Lin, 2012). ChIP PCR results showed that Gfi1aa could bind to these *etv2* regulator regions (*up1*, *-110 ∼ -35bp*, *intron-2*) compared to the gene body control region (*exon-8*) (Figures 3B,C), which is consistent with the ChIP-seq data (Figure 2E). Moreover, ChIP PCR also showed an enrichment of Gfi1aa on *sox7* regulatory region (*-520 ∼ 180bp*) (Figures 3D,E). These data suggest that the regulatory regions of *etv2* and *sox7* were directly bound by Gfi1aa.

As *etv2* and *sox7* are the master regulators of hematopoietic/endothelial cell differentiation, we examined whether *etv2* and *sox7* were the specific downstream target genes of Gfi1aa. We detected a series of hemangioblast markers—*scl*, *gata2*, and *fli1*, as well as *etv2* and *sox7*—at the beginning of primitive hematopoiesis. The results showed that *etv2* and *sox7* expression were markedly increased in *gfi1aa* ^*−/−*^ mutants compared to siblings, while expression of *scl, gata2,* and *fli1* was not altered (Supplementary Figure S5A). The expression of *etv2* and *sox7* by qPCR also showed a similar increase in *gfi1aa* ^*−/−*^ mutants compared to WT (Supplementary Figure S5B). The WISH and qPCR results verified the RNA-seq results that *etv2* and *sox7* are upregulated in *gfi1aa* ^*−/−*^ mutants.

We further performed reporter assays to determine whether Gfi1aa could repress *etv*2 and *sox7* transcription *in vivo*. We generated pTol-*etv2*-eGFP and pTol-*sox7*-eGFP reporter constructs and injected each construct into *gfi1aa* ^*+/-*^ intercross embryos to monitor whether GFP expression was affected by Gfi1aa (Figure 3F). The reporter assays showed that both *etv2*-eGFP and *sox7*-eGFP expression were increased in *gfi1aa* ^*−/−*^ mutants compared to their respective WT control (Figures 3G,H), suggesting a transcriptional repressive role for Gfi1aa in *etv2* and *sox7* regulatory regions.

The above data demonstrated that Gfi1aa targets the regulatory regions of *etv2* and *sox7* and suppresses their transcription.

### *sox7* and *etv2* Cooperatively Act Downstream of Gfi1aa for Hemangioblast Differentiation

We were eager to know whether downregulation of *sox7* rescued the blood deficiency of the *gfi1aa* ^*−/−*^ mutant. We injected *sox7* MO into *gfi1aa* ^*−/−*^ mutants and found that *alas2* ^+^ erythroid cell reduction and *flk1* ^*+*^ endothelial cell augmentation within the intermediate cell mass (ICM) region could be partially restored (Supplementary Figures S6A–D). It has been reported that *etv2* MO can also partially rescue *gfi1aa* mutant primitive hematopoietic defects (Moore et al., 2018). These data suggest that Gfi1aa targets not only *etv2* but also *sox7* to promote primitive erythrocyte differentiation from the hemangioblast.

**FIGURE 4:**
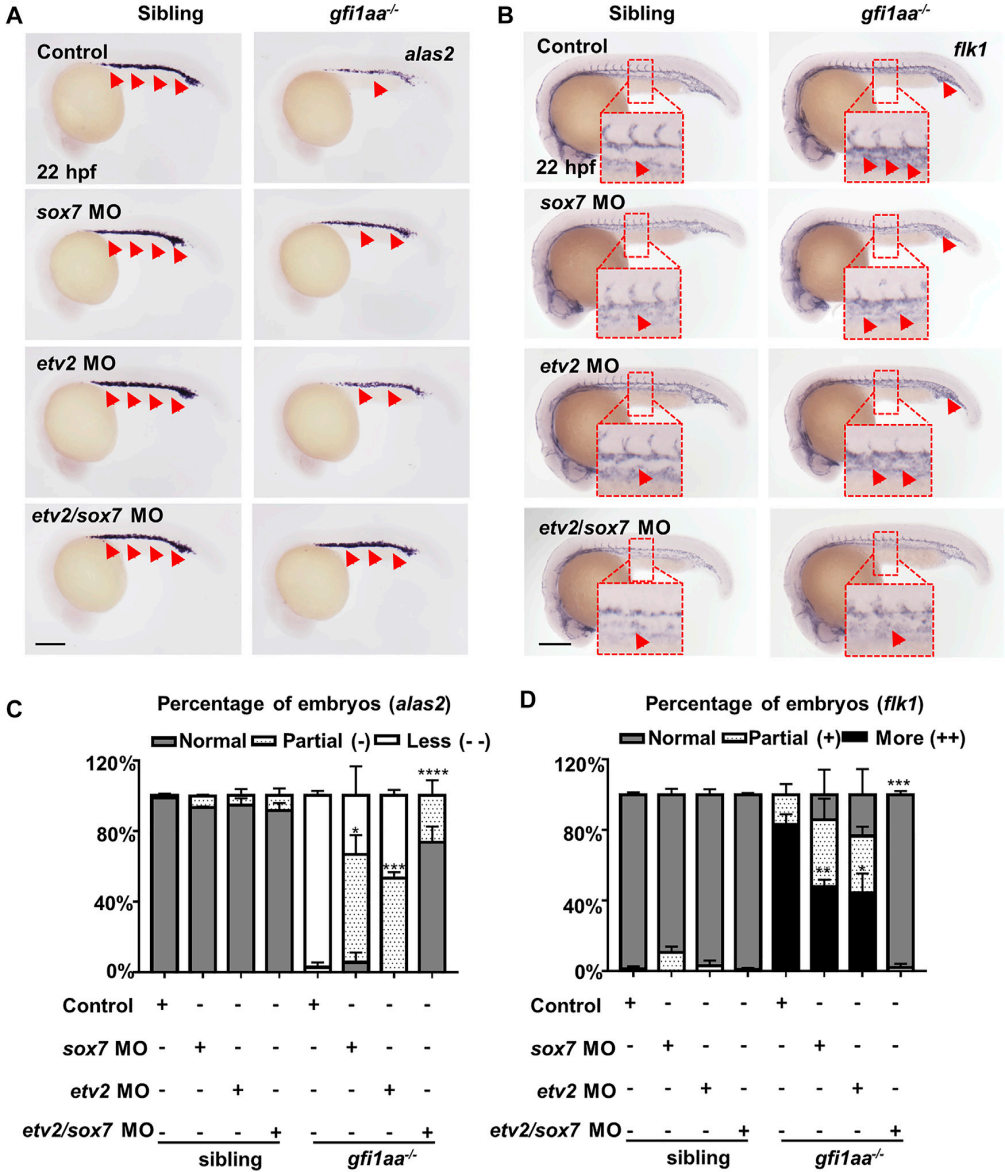
*sox7* and *etv2* act cooperatively to rescue the hematopoietic defect of *gfi1aa* mutant **(A, B)** Expression of *alas2* **(A)** and *flk1* **(B)** in siblings and *gfi1aa* ^*−/−*^ mutants injected with 0.5 pmol *sox7* MO, 0.005 pmol *etv2* MO, 0.5 pmol *sox7* MO with 0.005 pmol *etv2* MO or control. The red arrows indicated WISH signals and the red boxes indicated the magnification of ICM region. Scale bar: 200 μm **(C,D)** Analysis of *alas2* **(C)** and *flk1* **(D)** expression in siblings and *gfi1aa* ^*−/−*^ mutants rescued by *sox7* MO, *etv2* MO and *sox7* MO with *etv2* MO. The asterisks indicate the statistical difference of the rescued proportion by MO compared to *gfi1aa* ^*−/−*^ (Three independent experiments were performed, ****p < 0.0001, ***p < 0.001, **p < 0.01, *p < 0.05, *t*-test, n ≥ 10 embryos for each group).

Given the fact that either *etv2* or *sox7* partially rescued the primitive erythrocytes of the *gfi1aa* mutant, we speculated that *sox7* might cooperate with *etv2* for Gfi1aa regulated primitive erythropoiesis. To test this hypothesis, we knocked down both genes in *gfi1aa* ^*−/−*^ mutants to see if the hemangioblast differentiation defect could be further rescued. As a high dosage of *etv2* MO could cause severe vasculature defects of developing embryos (Sumanas and Lin, 2006), the cooperative effect on endothelial cells between *etv2* MO and *sox7* MO would be masked. Owing to this, we decreased *etv2* MO concentration and found 0.01 pmol *etv2* MO was enough to partially rescue the erythroid defect in *gfi1aa* mutant but not affect the vasculature which concentration was comparable to *sox7* MO (Supplementary Figures S7A–D). We therefore utilized the low dosage e*tv2* MO to involve in the double knockdown. Results showed that *alas2* ^+^ erythroid cell reduction and *flk1+* endothelial cells augmentation in *gfi1aa* ^*−/−*^ mutants could be almost completely restored (Figures 4A–D). These data suggest that the two transcription factors, *sox7* and *etv2*, act cooperatively downstream of Gfi1aa during hemangioblast differentiation.

### Gfi1aa Depends on Lsd1 to Repress *etv2* and *sox7* During Primitive Hemangioblast Differentiation

**FIGURE 5:**
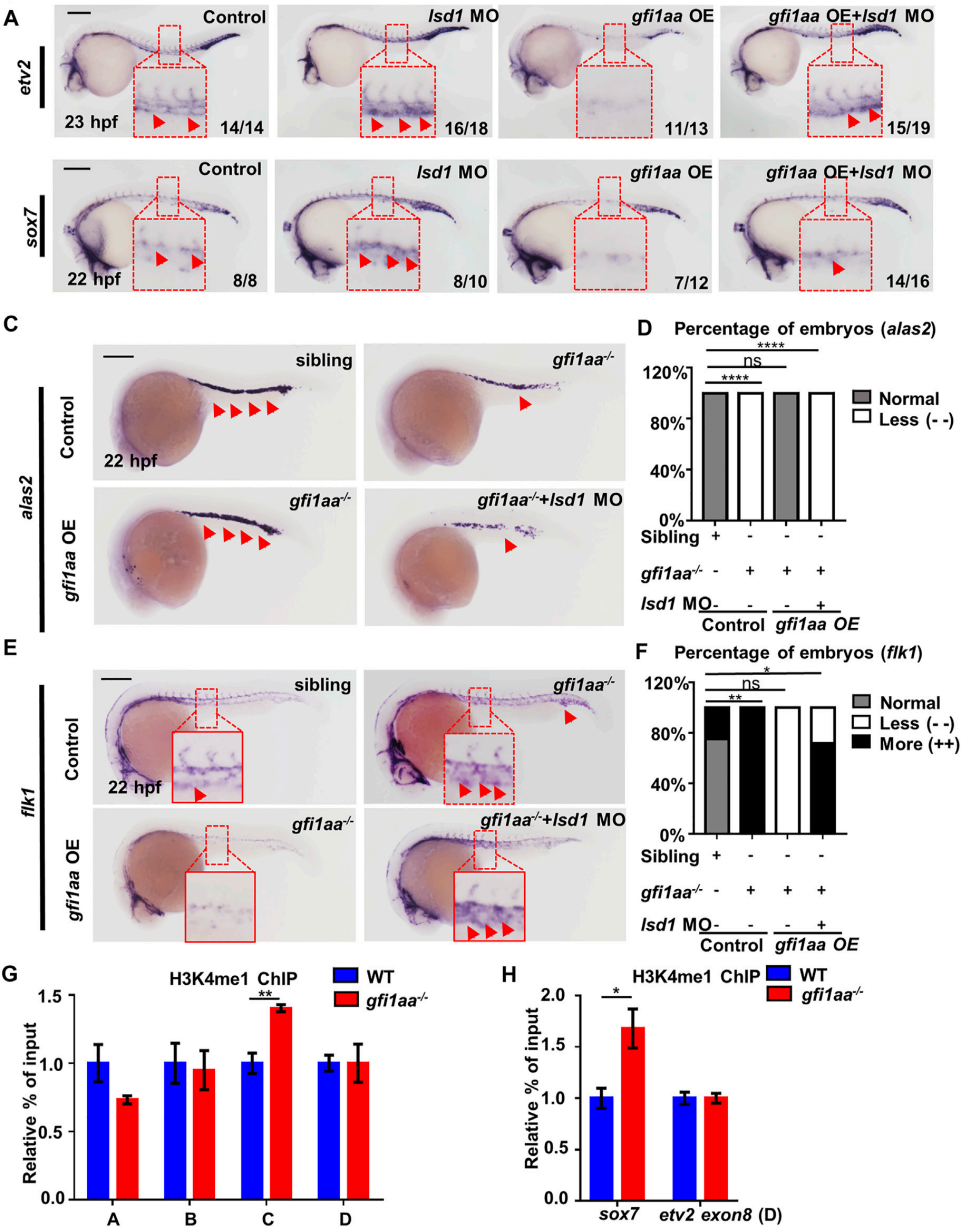
Gfi1aa targets *etv2 and sox7* in an Lsd1-dependent manner **(A,B)** Gfi1 repression activity on *etv2* and *sox7* transcription depends on Lsd1. Expression of *etv2* **(A)** and *sox7* **(B)** in WT, *lsd1* MO, *gfi1aa* overexpression (*gfi1aa*-OE) embryos, and *gfi1aa*-OE embryos co-injected with 1 pmol *lsd1* MO. *gfi1aa*-OE embryos were the progenies of *hsp-gfi1aa-eGFP* transgenic fish. The red boxes indicate the magnification of *etv2* signals **(A)** and *sox7* signals **(B)** in the ICM region. n ≥ 10 embryos for each group. The numbers in the bottom right corner indicate the percentage of embryos exhibiting the representative expression of indicated genes. Scale bar: 200 μm **(C,D)** Expression **(C)** and analysis **(D)** of erythroid marker *alas2* in sibling, *gfi1aa* ^*−/−*^ mutant, *gfi1aa-*OE rescued *gfi1aa* ^*−/−*^ mutant and *gfi1aa* ^*−/−*^ mutant with *gfi1aa-*OE and *lsd1*-MO at 22 hpf **(E,F)** Expression **(E)** and analysis **(F)** of endothelial marker *flk1* in sibling, *gfi1aa* ^*−/−*^ mutant, *gfi1aa-*OE rescued *gfi1aa* ^*−/−*^ mutant and *gfi1aa* ^*−/−*^ mutant with *gfi1aa-*OE and *lsd1*-MO at 22 hpf. The red boxes indicate the magnification of ICM region, and the red arrows indicate WISH signals (****p < 0.0001, **p < 0.01, *p < 0.05, ns, no significant, Fisher exact tests, n ≥ 10 embryos for each group). Scale bar: 200 μm **(G,H)** H3K4me1 levels *at etv2 intron-2* locus and *sox7* promoter were inhibited by Gfi1aa. ChIP-qPCR showed H3K4me1 level at *etv2* gene loci **(G)** and *sox7* promoter **(H)** in AB and *gfi1aa* ^*−/−*^ mutant embryos (The error bars represent three technical replicates and two independent experiments were performed, mean ± SEM; **p < 0.01; *t*-test).

As *lsd1*-deficient zebrafish (Takeuchi et al., 2015) phenocopied *gfi1aa* ^*−/−*^ mutants during primitive hematopoiesis and Gfi1aa could interact with Lsd1 in zebrafish (Wu et al., 2021), we speculated that Gfi1aa regulated hemangioblast differentiation into primitive erythrocytes was dependent upon Lsd1. We first inhibited *lsd1* to assess Gfi1aa repression of *etv2* and *sox7*, and found that the repression was indeed dependent on *lsd1*. Inhibited *etv2* and *sox7* expression levels in *gfi1aa*-overexpressing (*gfi1aa*-OE) embryos were rescued by downregulating *lsd1* (Figures 5A,B). This suggests that Gfi1aa requires Lsd1 to function as a transcriptional repressor. Furthermore, *gfi1aa*-OE rescued decreased *alas2* and increased *flk1* in *gfi1aa* ^*−/−*^ mutants, but downregulation of *lsd1* in *gfi1aa*-OE *gfi1aa* ^*−/−*^ mutants showed similar expression patterns to *gfi1aa* ^*−/−*^ mutants so that counteracted the restoration by *gfi1aa*-OE (Figures 5C–F), suggesting that Gfi1aa requires Lsd1 to function in promotion of hemangioblast differentiation into the primitive erythroid lineage.

Lsd1 is a histone demethylase that has been shown to repress *etv2* by alteration of associated H3K4 methylation during zebrafish primitive hematopoiesis (Takeuchi et al., 2015). Therefore, H3K4 methylation of *etv2* and *sox7* in *gfi1aa* ^*−/−*^ was assessed. The results showed H3K4me1 levels (primed and active enhancers marker (Heintzman et al., 2007; (Mercer et al., 2011)) to be upregulated in the regulatory regions of the two genes in *gfi1aa* ^*−/−*^ mutants (Figures 5G,H), suggesting that Gfi1aa and Lsd1 downregulate *etv2* and *sox7* by suppressing their H3K4me1 levels.

The above data demonstrate Gfi1aa to depend on Lsd1 to repress downstream *etv2* and *sox7* by altering H3K4 methylation during primitive hemangioblast differentiation.

## Discussion

**FIGURE 6:**
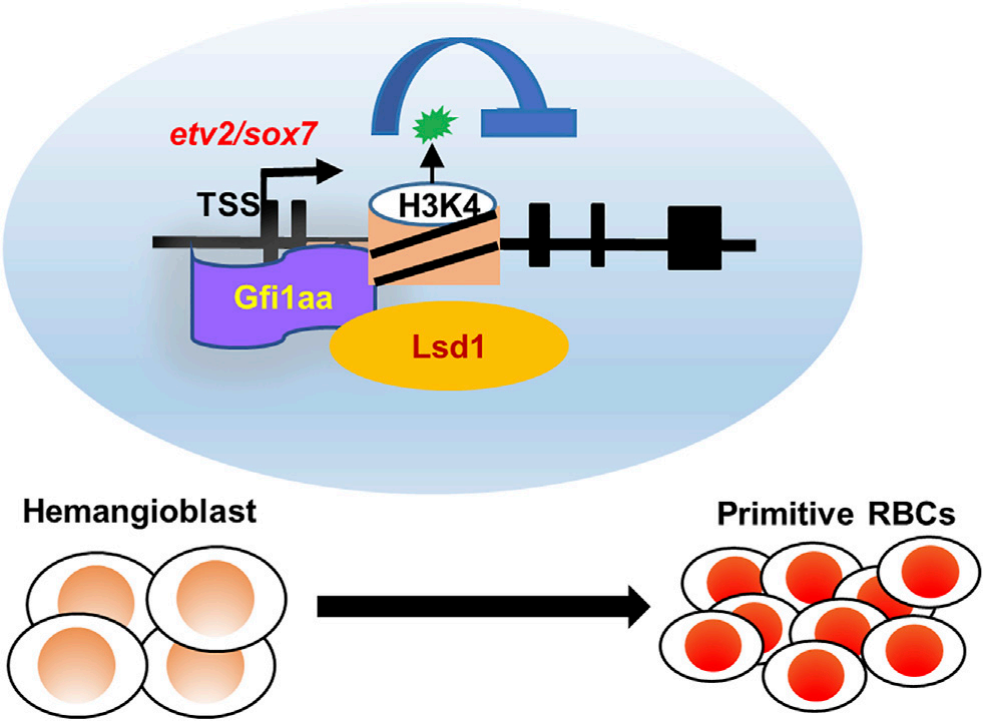
Working model of Gfi1aa/Lsd1-*etv2/sox7* in primitive erythropoiesis. Gfi1aa/Lsd1-*etv2/sox7* regulatory modules in hemangioblast differentiation into primitive red blood cells.

In this study, we demonstrated complex roles for *gfi1(s)* in primitive erythropoiesis by genetic analysis of *gfi1* single, double, and triple mutants. We revealed that *gfi1aa* played a predominant role in regulating hemangioblast differentiation, and *gfi1ab*, similar to *gfi1b*, played a compensatory role. Further, by bioinformatics assays and genetic analysis, we identified *sox7* and *etv2* as two key downstream targets of Gfi1aa, as Gfi1aa directly bound to the regulatory regions of the two transcription factors and suppressed their expression. Gfi1aa suppressed downstream target expressions in an Lsd1-dependent manner by altering their H3K4 methylation status. The study reveals that the Gfi1aa/Lsd1-dependent *etv2* and *sox7* suppression facilitates hemangioblast differentiation into primitive erythrocytes (Figure 6), which provides new insights into the generation of the first blood cells.

In mammals, both Gfi1 and Gfi1b are major regulators of hematopoiesis (Hock and Orkin, 2006; (van der Meer et al., 2010; (Moroy et al., 2015). Gfi1 is mainly involved in HSC self-renewal (Hock et al., 2004; (Zeng et al., 2004), lymphoid development (Yucel et al., 2003), and neutrophil differentiation (Hock et al., 2003), whereas Gfi1b is required for erythropoiesis (Saleque et al., 2002). GFI1B can compensate for GFI1 function in definitive hematopoiesis when GFI1 has lost function (Fiolka et al., 2006). Zebrafish has three Gfi1 members: Gfi1aa, Gfi1ab, and Gfi1b. By genetic analysis of *gfi1* single, double, and triple mutants, we demonstrated complex roles for *gfi1(s)* in primitive erythropoiesis. We generated a *gfi1ab* ^*−/−*^ mutant which showed no hematopoietic defect. It is reported that *gfi1ab* is ectopically expressed in the ICM region of *gfi1aa* ^*qmc551*^ mutants (Thambyrajah et al., 2016b; (Moore et al., 2018), our WISH further showed it expressed in the ICM region of all *gfi1aa*-related mutants, suggesting its compensatory role for *gfi1aa* function. With genetic evidence, we found that *gfi1aa*-related double and triple mutants have severe defects in primitive erythropoiesis. We hence concluded that *gfi1aa* played a predominant role, and *gfi1ab*, similar to *gfi1b*, played a compensatory role in regulating hemangioblast differentiation. Our results suggest differing and redundant roles for three *gfi1* members in hematopoiesis.

Both *Etv2* and *Sox7* are hemangioblast markers that control hematopoietic and endothelial cell emergence (Gandillet et al., 2009; (Kataoka et al., 2011; (Costa et al., 2012; (Sumanas and Choi, 2016). Knockdown of *Sox7* reduced both hematopoietic and endothelial cells (Gandillet et al., 2009; (Costa et al., 2012), whereas its overexpression increased endothelial markers (Costa et al., 2012). Similarly, *Etv2-*deficient mice (Lee et al., 2008) and *etv2* zebrafish mutants (Pham et al., 2007) displayed both blood and endothelial cells disruption, while enforced expression of *etv2* resulted in persistent endothelial specification (Sumanas and Lin, 2006; (Hayashi et al., 2012). Herein, we demonstrated both *etv2* and *sox7* to be upregulated in all *gfi1aa*-related mutants, while downregulation of the genes rescued the hematopoietic defect in the *gfi1aa* ^*−/−*^ mutant. Notably, both genes were directly targeted and suppressed by Gfi1aa in an *lsd1*-dependent manner. In previously reported *lsd1* zebrafish mutant, *etv2* is upregulated, and when downregulated, it rescues the hematopoietic defect of *lsd1* mutants (Takeuchi et al., 2015). Moreover, *lsd1* MO and *gfi1aa* ^*−/−*^ mutant exhibited a similar increase of H3K4me1 status at *etv2* intron2, suggesting the co-regulation of Gfi1aa and Lsd1 on *etv2*. Our genetic and molecular analysis demonstrated the likely interplay among Gfi1aa, Lsd1, as well as *sox7* and *etv2* during primitive hematopoiesis. At the onset of primitive hematopoiesis, Gfi1aa/Lsd1 inhibits *etv2* and *sox7* by preventing maintenance of the endothelial characteristics of hemangioblasts. *etv2* and *sox7*, repressed by Gfi1aa and Lsd1 cooperation, synergistically control hemangioblast differentiation. We further knocked down *etv2* and *sox7* in *gfi1aa/1b* ^*DM*^ and *gfi1* ^*TM*^ mutants, whereas *etv2*/*sox7* MO partially restored the *alas2* ^+^ erythroid cells and *flk1* ^*+*^ endothelial cells in these mutants (Supplementary Figures 8A–D), suggesting *etv2* and *sox7* are indeed the targets of Gfi1aa whereas other factors (e.g., *flk1*, *cdh5,* and *egfl7*) or pathways involve in hematopoiesis regulation remain further investigation.

During the definitive wave, hematopoietic stem cells (HSC) are derived from the hemogenic endothelium (HE) in the ventral wall of the dorsal aorta (VDA) by a process of endothelial to hematopoietic transition (EHT) (Bertrand et al., 2010). HSC-forming HE was derived from the arterial endothelium (Bonkhofer et al., 2019). For mouse embryonic HSC development, GFI1 and GFI1B, which are regulated by RUNX1 (Lancrin et al., 2012), inhibit endothelial programs to facilitate the EHT process of HSC development by recruiting the chromatin remodeler LSD1 (Thambyrajah et al., 2016a). Here, we demonstrated that Gfi1aa is dependent on Lsd1 for transcriptional suppression of endothelial factors in hemangioblast differentiation to primitive hematopoiesis. Based on current knowledge, the initial developmental processes for primitive and definitive hematopoiesis seem similar, as hematopoietic cells in two waves are both derived from bi-potential (or multi-potential) progenitors with potent endothelial specification. Since Gfi1/Lsd1 suppresses endothelial specification in both definitive and primitive waves, this suggests the regulatory module of Gfi1/Lsd1 might be a confluent of the two distinct hematopoietic waves, which may be conserved across species. It is possible that primitive hematopoietic cells, derived from hemangioblasts, share a similar molecular progression to the definitive wave of EHT. Thus, the distinct hematopoiesis waves may converge to the Gfi1(s)/Lsd1 module or even Gfi1(s)/Lsd1-*etv2/sox7* involved molecular regulatory pathway.

Taken together, the results of our study demonstrate that the regulatory module Gfi1aa-Lsd1-*etv2/sox7* plays a pivotal role in downregulating endothelial genes to promote hemangioblast differentiation into primitive erythrocytes. These results elucidate the genetic and epigenetic regulatory mechanisms of Gfi1(s) on the process of how primitive hematopoiesis begins with hemangioblasts. Since Gfi1/Lsd1 suppresses endothelial specification of both definitive and primitive waves, it suggests the regulatory module of Gfi1/Lsd1 might be a confluent of the two distinct hematopoietic waves. Thus, both hematopoiesis waves may converge to the Gfi1(s)/Lsd1 involved molecular regulatory pathway.

## Data Availability

The datasets presented in this study can be found in online repositories. The names of the repository/repositories and accession number(s) can be found below: https://www.ncbi.nlm.nih.gov/, GSE181395.

## References

[B1] Baron M. H. Isern J. Fraser S. T. (2012). The Embryonic Origins of Erythropoiesis in Mammals. Blood 119, 4828–4837. 10.1182/blood-2012-01-153486 22337720 PMC3367890

[B2] Bertrand J. Y. Chi N. C. Santoso B. Teng S. Stainier D. Y. R. Traver D. (2010). Haematopoietic Stem Cells Derive Directly from Aortic Endothelium during Development. Nature 464, 108–111. 10.1038/nature08738 20154733 PMC2858358

[B3] Bonkhofer F. Rispoli R. Pinheiro P. Krecsmarik M. Schneider-Swales J. Tsang I. H. C. (2019). Blood Stem Cell-Forming Haemogenic Endothelium in Zebrafish Derives from Arterial Endothelium. Nat. Commun. 10, 3577. 10.1038/s41467-019-11423-2 31395869 PMC6687740

[B4] Cermenati S. Moleri S. Cimbro S. Corti P. Del Giacco L. Amodeo R. (2008). Sox18 and Sox7 Play Redundant Roles in Vascular Development. Blood 111, 2657–2666. 10.1182/blood-2007-07-100412 18094332

[B5] Chang N. Sun C. Gao L. Zhu D. Xu X. Zhu X. (2013). Genome Editing with RNA-Guided Cas9 Nuclease in Zebrafish Embryos. Cell Res 23, 465–472. 10.1038/cr.2013.45 23528705 PMC3616424

[B6] Chen A. T. Zon L. I. (2009). Zebrafish Blood Stem Cells. J. Cel. Biochem. 108, 35–42. 10.1002/jcb.22251 19565566

[B7] Cooney J. D. Hildick-Smith G. J. Shafizadeh E. McBride P. F. Carroll K. J. Anderson H. (2013). Teleost Growth Factor independence (Gfi) Genes Differentially Regulate Successive Waves of Hematopoiesis. Developmental Biol. 373, 431–441. 10.1016/j.ydbio.2012.08.015 PMC3532562 22960038

[B8] Costa G. Mazan A. Gandillet A. Pearson S. Lacaud G. Kouskoff V. (2012). SOX7 Regulates the Expression of VE-Cadherin in the Haemogenic Endothelium at the Onset of Haematopoietic Development. Development 139, 1587–1598. 10.1242/dev.071282 22492353

[B9] Dobin A. Davis C. A. Schlesinger F. Drenkow J. Zaleski C. Jha S. (2013). STAR: Ultrafast Universal RNA-Seq Aligner. Bioinformatics 29, 15–21. 10.1093/bioinformatics/bts635 23104886 PMC3530905

[B10] Dufourcq P. Rastegar S. Strähle U. Blader P. (2004). Parapineal Specific Expression of Gfi1 in the Zebrafish Epithalamus. Gene Expr. Patterns 4, 53–57. 10.1016/s1567-133x(03)00148-0 14678828

[B11] Fiolka K. Hertzano R. Vassen L. Zeng H. Hermesh O. Avraham K. B. (2006). Gfi1 and Gfi1b Act Equivalently in Haematopoiesis, but Have Distinct, Non‐overlapping Functions in Inner Ear Development. EMBO Rep. 7, 326–333. 10.1038/sj.embor.7400618 16397623 PMC1456886

[B12] Gandillet A. Serrano A. G. Pearson S. Lie-A-Ling M. Lacaud G. Kouskoff V. (2009). Sox7-sustained Expression Alters the Balance between Proliferation and Differentiation of Hematopoietic Progenitors at the Onset of Blood Specification. Blood 114, 4813–4822. 10.1182/blood-2009-06-226290 19801444

[B13] Garcia M. D. Larina I. V. (2014). Vascular Development and Hemodynamic Force in the Mouse Yolk Sac. Front. Physiol. 5, 308. 10.3389/fphys.2014.00308 25191274 PMC4138559

[B14] Gering M. Rodaway A. R. F. Göttgens B. Patient R. K. Green A. R. (1998). The SCL Gene Specifies Haemangioblast Development from Early Mesoderm. EMBO J. 17, 4029–4045. 10.1093/emboj/17.14.4029 9670018 PMC1170736

[B15] Hart A. Melet F. Grossfeld P. Chien K. Jones C. Tunnacliffe A. (2000). Fli-1 Is Required for Murine Vascular and Megakaryocytic Development and Is Hemizygously Deleted in Patients with Thrombocytopenia. Immunity 13, 167–177. 10.1016/s1074-7613(00)00017-0 10981960

[B16] Hayashi M. Pluchinotta M. Momiyama A. Tanaka Y. Nishikawa S.-I. Kataoka H. (2012). Endothelialization and Altered Hematopoiesis by Persistent Etv2 Expression in Mice. Exp. Hematol. 40, 738–750. e711. 10.1016/j.exphem.2012.05.012 22659386

[B17] Heintzman N. D. Stuart R. K. Hon G. Fu Y. Ching C. W. Hawkins R. D. (2007). Distinct and Predictive Chromatin Signatures of Transcriptional Promoters and Enhancers in the Human Genome. Nat. Genet. 39, 311–318. 10.1038/ng1966 17277777

[B18] Hock H. Hamblen M. J. Rooke H. M. Schindler J. W. Saleque S. Fujiwara Y. (2004). Gfi-1 Restricts Proliferation and Preserves Functional Integrity of Haematopoietic Stem Cells. Nature 431, 1002–1007. 10.1038/nature02994 15457180

[B19] Hock H. Hamblen M. J. Rooke H. M. Traver D. Bronson R. T. Cameron S. (2003). Intrinsic Requirement for Zinc finger Transcription Factor Gfi-1 in Neutrophil Differentiation. Immunity 18, 109–120. 10.1016/s1074-7613(02)00501-0 12530980

[B20] Hock H. Orkin S. H. (2006). Zinc-finger Transcription Factor Gfi-1: Versatile Regulator of Lymphocytes, Neutrophils and Hematopoietic Stem Cells. Curr. Opin. Hematol. 13, 1–6. 10.1097/01.moh.0000190111.85284.8f 16319680

[B21] Jin S.-W. Beis D. Mitchell T. Chen J.-N. Stainier D. Y. R. (2005). Cellular and Molecular Analyses of Vascular Tube and Lumen Formation in Zebrafish. Development 132, 5199–5209. 10.1242/dev.02087 16251212

[B22] Kaipainen A. Korhonen J. Mustonen T. van Hinsbergh V. W. Fang G. H. Dumont D. (1995). Expression of the Fms-like Tyrosine Kinase 4 Gene Becomes Restricted to Lymphatic Endothelium during Development. Proc. Natl. Acad. Sci. 92, 3566–3570. 10.1073/pnas.92.8.3566 7724599 PMC42208

[B23] Kataoka H. Hayashi M. Nakagawa R. Tanaka Y. Izumi N. Nishikawa S. (2011). Etv2/ER71 Induces Vascular Mesoderm from Flk1+PDGFRα+ Primitive Mesoderm. Blood 118, 6975–6986. 10.1182/blood-2011-05-352658 21911838

[B24] Lacaud G. Kouskoff V. (2017). Hemangioblast, Hemogenic Endothelium, and Primitive versus Definitive Hematopoiesis. Exp. Hematol. 49, 19–24. 10.1016/j.exphem.2016.12.009 28043822

[B25] Lancrin C. Mazan M. Stefanska M. Patel R. Lichtinger M. Costa G. (2012). GFI1 and GFI1B Control the Loss of Endothelial Identity of Hemogenic Endothelium during Hematopoietic Commitment. Blood 120, 314–322. 10.1182/blood-2011-10-386094 22668850

[B26] Lancrin C. Sroczynska P. Stephenson C. Allen T. Kouskoff V. Lacaud G. (2009). The Haemangioblast Generates Haematopoietic Cells through a Haemogenic Endothelium Stage. Nature 457, 892–895. 10.1038/nature07679 19182774 PMC2661201

[B27] Lee D. Park C. Lee H. Lugus J. J. Kim S. H. Arentson E. (2008). ER71 Acts Downstream of BMP, Notch, and Wnt Signaling in Blood and Vessel Progenitor Specification. Cell Stem Cell 2, 497–507. 10.1016/j.stem.2008.03.008 18462699 PMC2683414

[B28] Liu D. Wang Z. Xiao A. Zhang Y. Li W. Zu Y. (2014). Efficient Gene Targeting in Zebrafish Mediated by a Zebrafish-Codon-Optimized Cas9 and Evaluation of Off-Targeting Effect. J. Genet. Genomics 41, 43–46. 10.1016/j.jgg.2013.11.004 24480746

[B29] Liu F. Patient R. (2008). Genome-wide Analysis of the Zebrafish ETS Family Identifies Three Genes Required for Hemangioblast Differentiation or Angiogenesis. Circ. Res. 103, 1147–1154. 10.1161/CIRCRESAHA.108.179713 18832752

[B30] Liu F. Walmsley M. Rodaway A. Patient R. (2008). Fli1 Acts at the Top of the Transcriptional Network Driving Blood and Endothelial Development. Curr. Biol. 18, 1234–1240. 10.1016/j.cub.2008.07.048 18718762

[B31] Love M. I. Huber W. Anders S. (2014). Moderated Estimation of Fold Change and Dispersion for RNA-Seq Data with DESeq2. Genome Biol. 15, 550. 10.1186/s13059-014-0550-8 25516281 PMC4302049

[B32] Lugus J. J. Chung Y. S. Mills J. C. Kim S.-I. Grass J. A. Kyba M. (2007). GATA2 Functions at Multiple Steps in Hemangioblast Development and Differentiation. Development 134, 393–405. 10.1242/dev.02731 17166922

[B33] Mercer E. M. Lin Y. C. Benner C. Jhunjhunwala S. Dutkowski J. Flores M. (2011). Multilineage Priming of Enhancer Repertoires Precedes Commitment to the B and Myeloid Cell Lineages in Hematopoietic Progenitors. Immunity 35, 413–425. 10.1016/j.immuni.2011.06.013 21903424 PMC3183365

[B34] Moore C. Richens J. L. Hough Y. Ucanok D. Malla S. Sang F. (2018). Gfi1aa and Gfi1b Set the Pace for Primitive Erythroblast Differentiation from Hemangioblasts in the Zebrafish Embryo. Blood Adv. 2, 2589–2606. 10.1182/bloodadvances.2018020156 30309860 PMC6199651

[B35] Möröy T. Vassen L. Wilkes B. Khandanpour C. (2015). From Cytopenia to Leukemia: the Role of Gfi1 and Gfi1b in Blood Formation. Blood 126, 2561–2569. 10.1182/blood-2015-06-655043 26447191 PMC4671106

[B36] Murray P. (1932). The Development *In Vitro* of the Blood of the Early Chick Embryo. Proc. R. Soc. Lond. B. 111, 497–521. 10.1098/rspb.1932.0070

[B37] Parker L. H. Schmidt M. Jin S.-W. Gray A. M. Beis D. Pham T. (2004). The Endothelial-Cell-Derived Secreted Factor Egfl7 Regulates Vascular Tube Formation. Nature 428, 754–758. 10.1038/nature02416 15085134

[B38] Patterson L. J. Gering M. Eckfeldt C. E. Green A. R. Verfaillie C. M. Ekker S. C. (2007). The Transcription Factors Scl and Lmo2 Act Together during Development of the Hemangioblast in Zebrafish. Blood 109, 2389–2398. 10.1182/blood-2006-02-003087 17090656

[B39] Pham V. N. Lawson N. D. Mugford J. W. Dye L. Castranova D. Lo B. (2007). Combinatorial Function of ETS Transcription Factors in the Developing Vasculature. Developmental Biol. 303, 772–783. 10.1016/j.ydbio.2006.10.030 PMC1859867 17125762

[B40] Saleque S. Cameron S. Orkin S. H. (2002). The Zinc-finger Proto-Oncogene Gfi-1b Is Essential for Development of the Erythroid and Megakaryocytic Lineages. Genes Dev. 16, 301–306. 10.1101/gad.959102 11825872 PMC155332

[B41] Saleque S. Kim J. Rooke H. M. Orkin S. H. (2007). Epigenetic Regulation of Hematopoietic Differentiation by Gfi-1 and Gfi-1b Is Mediated by the Cofactors CoREST and LSD1. Mol. Cel 27, 562–572. 10.1016/j.molcel.2007.06.039 17707228

[B42] Spyropoulos D. D. Pharr P. N. Lavenburg K. R. Jackers P. Papas T. S. Ogawa M. (2000). Hemorrhage, Impaired Hematopoiesis, and Lethality in Mouse Embryos Carrying a Targeted Disruption of the Fli1 Transcription Factor. Mol. Cel Biol 20, 5643–5652. 10.1128/mcb.20.15.5643-5652.2000 PMC86032 10891501

[B43] Sumanas S. Choi K. (2016). ETS Transcription Factor ETV2/ER71/Etsrp in Hematopoietic and Vascular Development. Curr. Top. Dev. Biol. 118, 77–111. 10.1016/bs.ctdb.2016.01.005 27137655

[B44] Sumanas S. Jorniak T. Lin S. (2005). Identification of Novel Vascular Endothelial-specific Genes by the Microarray Analysis of the Zebrafish Cloche Mutants. Blood 106, 534–541. 10.1182/blood-2004-12-4653 15802528 PMC1895181

[B45] Sumanas S. Lin S. (2006). Ets1-related Protein Is a Key Regulator of Vasculogenesis in Zebrafish. Plos Biol. 4, e10. 10.1371/journal.pbio.0040010 16336046 PMC1310653

[B46] Takeuchi M. Fuse Y. Watanabe M. Andrea C.-S. Takeuchi M. Nakajima H. (2015). LSD1/KDM1A Promotes Hematopoietic Commitment of Hemangioblasts through Downregulation of Etv2. Proc. Natl. Acad. Sci. USA 112, 13922–13927. 10.1073/pnas.1517326112 26512114 PMC4653156

[B47] Thambyrajah R. Mazan M. Patel R. Moignard V. Stefanska M. Marinopoulou E. (2016a). GFI1 Proteins Orchestrate the Emergence of Haematopoietic Stem Cells through Recruitment of LSD1. Nat. Cel Biol 18, 21–32. 10.1038/ncb3276 26619147

[B48] Thambyrajah R. Ucanok D. Jalali M. Hough Y. Wilkinson R. N. McMahon K. (2016b). A Gene Trap Transposon Eliminates Haematopoietic Expression of Zebrafish Gfi1aa, but Does Not Interfere with Haematopoiesis. Developmental Biol. 417, 25–39. 10.1016/j.ydbio.2016.07.010 PMC5003831 27432513

[B49] Thisse C. Thisse B. (2008). High-resolution *In Situ* Hybridization to Whole-Mount Zebrafish Embryos. Nat. Protoc. 3, 59–69. 10.1038/nprot.2007.514 18193022

[B50] van der Meer L. T. Jansen J. H. van der Reijden B. A. (2010). Gfi1 and Gfi1b: Key Regulators of Hematopoiesis. Leukemia 24, 1834–1843. 10.1038/leu.2010.195 20861919

[B51] Veldman M. B. Lin S. (2012). Etsrp/Etv2 Is Directly Regulated by Foxc1a/b in the Zebrafish Angioblast. Circ. Res. 110, 220–229. 10.1161/CIRCRESAHA.111.251298 22135404 PMC3457812

[B52] Velinder M. Singer J. Bareyan D. Meznarich J. Tracy C. M. Fulcher J. M. (2016). GFI1 Functions in Transcriptional Control and Cell Fate Determination Require SNAG Domain Methylation to Recruit LSD1. Biochem. J. 473, 3355–3369. 10.1042/BCJ20160558 27480105

[B53] Vogeli K. M. Jin S.-W. Martin G. R. Stainier D. Y. R. (2006). A Common Progenitor for Haematopoietic and Endothelial Lineages in the Zebrafish Gastrula. Nature 443, 337–339. 10.1038/nature05045 16988712

[B54] Wei W. Wen L. Huang P. Zhang Z. Chen Y. Xiao A. (2008). Gfi1.1 Regulates Hematopoietic Lineage Differentiation during Zebrafish Embryogenesis. Cel Res 18, 677–685. 10.1038/cr.2008.60 18504458

[B55] Westerfield M. (2000). The Zebrafish Book. A Guide for the Laboratory Use of Zebrafish (*Danio rerio*). 4th ed. Eugene: Univ. of Oregon Press.

[B56] Wu M. Xu Y. Li J. Lian J. Chen Q. Meng P. (2021). Genetic and Epigenetic Orchestration of Gfi1aa-Lsd1-Cebpα in Zebrafish Neutrophil Development. Development 148 (17), dev199516. 10.1242/dev.199516 34373913

[B57] Yücel R. Karsunky H. Klein-Hitpass L. Möröy T. (2003). The Transcriptional Repressor Gfi1 Affects Development of Early, Uncommitted C-Kit+ T Cell Progenitors and CD4/CD8 Lineage Decision in the Thymus. J. Exp. Med. 197, 831–844. 10.1084/jem.20021417 12682108 PMC2193890

[B58] Zeng H. Yücel R. Kosan C. Klein-Hitpass L. Möröy T. (2004). Transcription Factor Gfi1 Regulates Self-Renewal and Engraftment of Hematopoietic Stem Cells. EMBO J. 23, 4116–4125. 10.1038/sj.emboj.7600419 15385956 PMC524350

[B59] Zhou Y. Zhou B. Pache L. Chang M. Khodabakhshi A. H. Tanaseichuk O. (2019). Metascape Provides a Biologist-Oriented Resource for the Analysis of Systems-Level Datasets. Nat. Commun. 10, 1523. 10.1038/s41467-019-09234-6 30944313 PMC6447622

