# Supplementary material for: Gfi1aa/Lsd1 Facilitates Hemangioblast Differentiation Into Primitive Erythrocytes by Targeting etv2 and sox7 in Zebrafish
Source: Front Cell Dev Biol. 2022 Jan 12;9:786426. doi: 10.3389/fcell.2021.786426 (PMC8790037; doi:10.3389/fcell.2021.786426)
Supplement: Supplementary file 1 [file Table1.DOCX]

**Supplementary Table 1. Primers for PCR**

| **Primer** | **Forward (5’-3’)** | **Reverse (5’-3’)** |
| --- | --- | --- |
| *gfi1b* | GTTCATGCTAACTCAGTGCATAC | TCTGAACTCCTGATATGAGGC |
| *gfi1ab* | CACCCTCCGTGGAAGAAACAC | GCGCCATTTAACAGGAGACGA |
| *etv2* (qPCR) | ctacccaggatctggaccca | tttaaactcccagccatcacca |
| *sox7* (qPCR) | tcaggagacccatgaacgcc | cagcagcctctaggatccggaa |
| *sox7* (ChIP-PCR) | tgtgctggaagctgctcctc | ggcgcagaggcgacctatag |
| *etv2* (construct) | ccgCTCGAGCTGTAGACATGGGCGTGCGC | CGGGATCCcggcatactgctgttggacgg |
| *sox7* (construct) | ccgCTCGAGccactaaacaatacgtacagtg | cgGGATCCtgtgcgctgtcaaaacttag |
