# Supplementary material for: Gfi1aa/Lsd1 Facilitates Hemangioblast Differentiation Into Primitive Erythrocytes by Targeting etv2 and sox7 in Zebrafish
Source: Front Cell Dev Biol. 2022 Jan 12;9:786426. doi: 10.3389/fcell.2021.786426 (PMC8790037; doi:10.3389/fcell.2021.786426)
Supplement: Supplementary file 2 [file DataSheet1.pdf]

Supplementary Figures

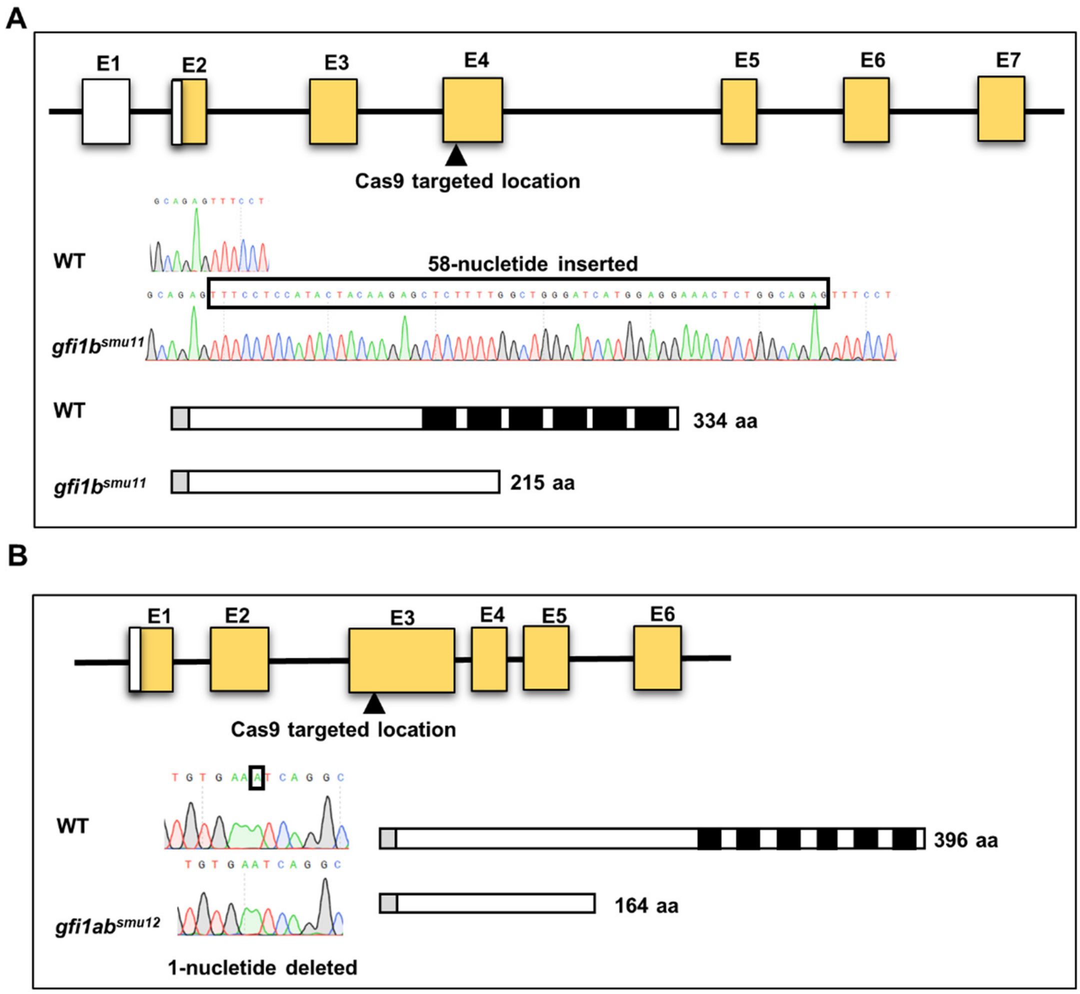

Supplementary Figure 1. Generation of *gfi1b*^{smu11} and *gfi1ab*^{smu12} mutant. (A) *gfi1b*^{smu11} mutant was generated by CRISPR/Cas9. *gfi1b* Cas9 gRNA targeted at exon4 and a 58-nucleotide insertion generated, consequently the truncated protein generated in *gfi1b*^{smu11} mutant. **(B)** *gfi1ab*^{smu12} mutant was generated by CRISPR/Cas9. *gfi1ab* Cas9 gRNA targeted at exon3 and a 1-nucleotide deletion generated, consequently the truncated protein generated in *gfi1ab*^{smu12} mutant. For Gfi1b and Gfi1ab protein structure, the gray-boxed region indicates the SNAG domain and the black-boxed regions indicate the DNA binding domain.

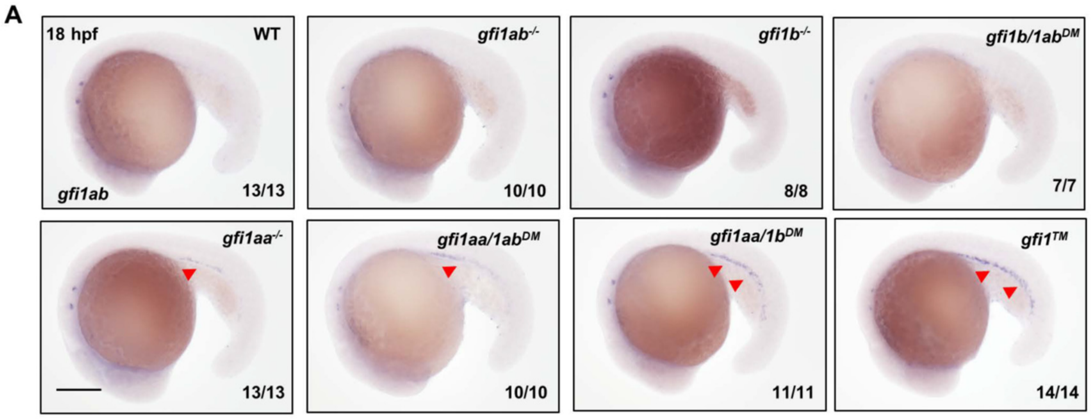

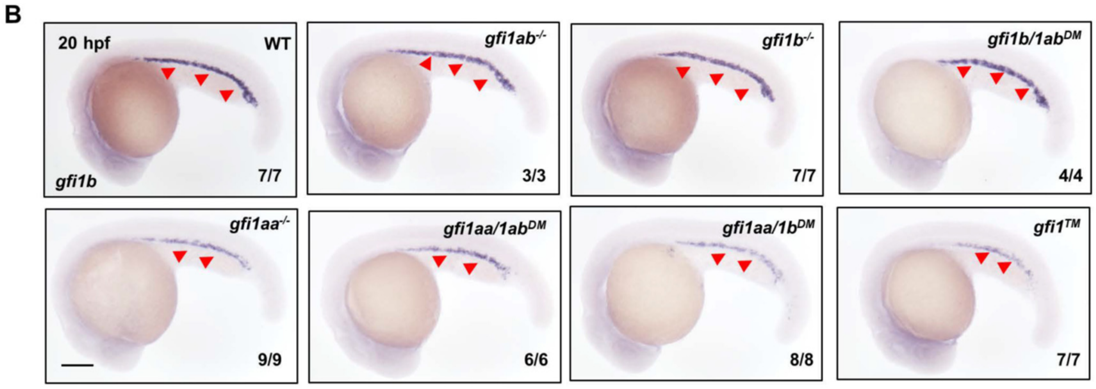

Supplementary Figure 2. *gfi1ab* and *gfi1b* expression among *gfi1* mutants. Expression of *gfi1ab* (A) and *gfi1b* (B) in WT, *gfi1aa*$^{-/-}$, *gfi1b*$^{-/-}$, *gfi1ab*$^{-/-}$ signal mutant, *gfi1aa/1b*^{DM}, *gfi1aa/1ab*^{DM}, *gfi1b/1ab*^{DM} double mutant and *gfi1*^{TM} triple mutant by WISH. The numbers in the bottom right corner indicate representative expression embryo numbers of the indicated marker. Scale bar: 200 μm.

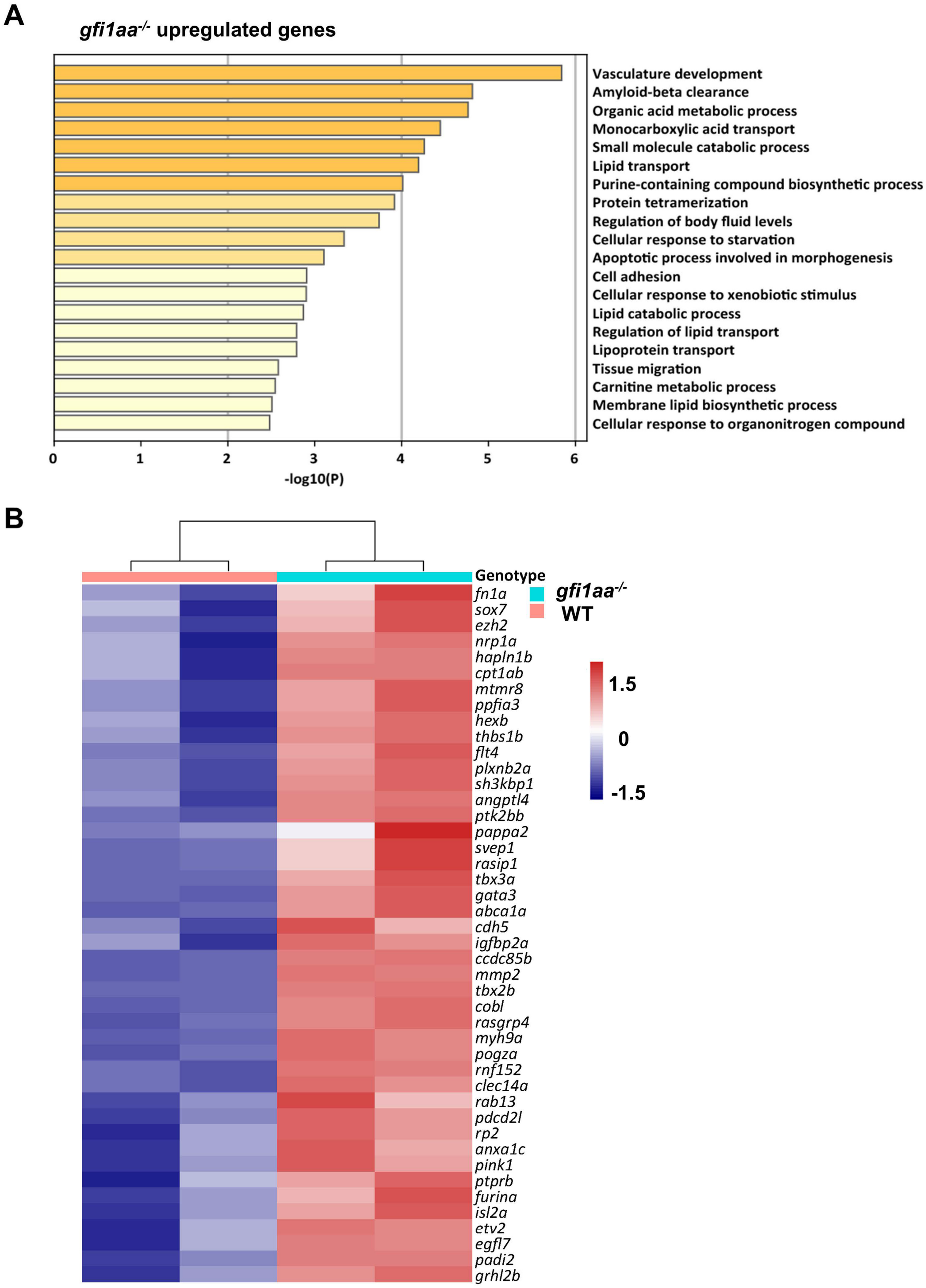

Supplementary Figure 3. Vasculature markers were increased in *gfi1aa*-related mutants. (A) Go enrichment analysis of the up-regulated gene in *gfi1aa*$^{-/-}$ mutant RNA-seq. **(B)** Heat map of WT and *gfi1aa*$^{-/-}$ mutant showed the vasculature development genes expression levels from **(A)**. The color scale illustrated the gene expression levels.

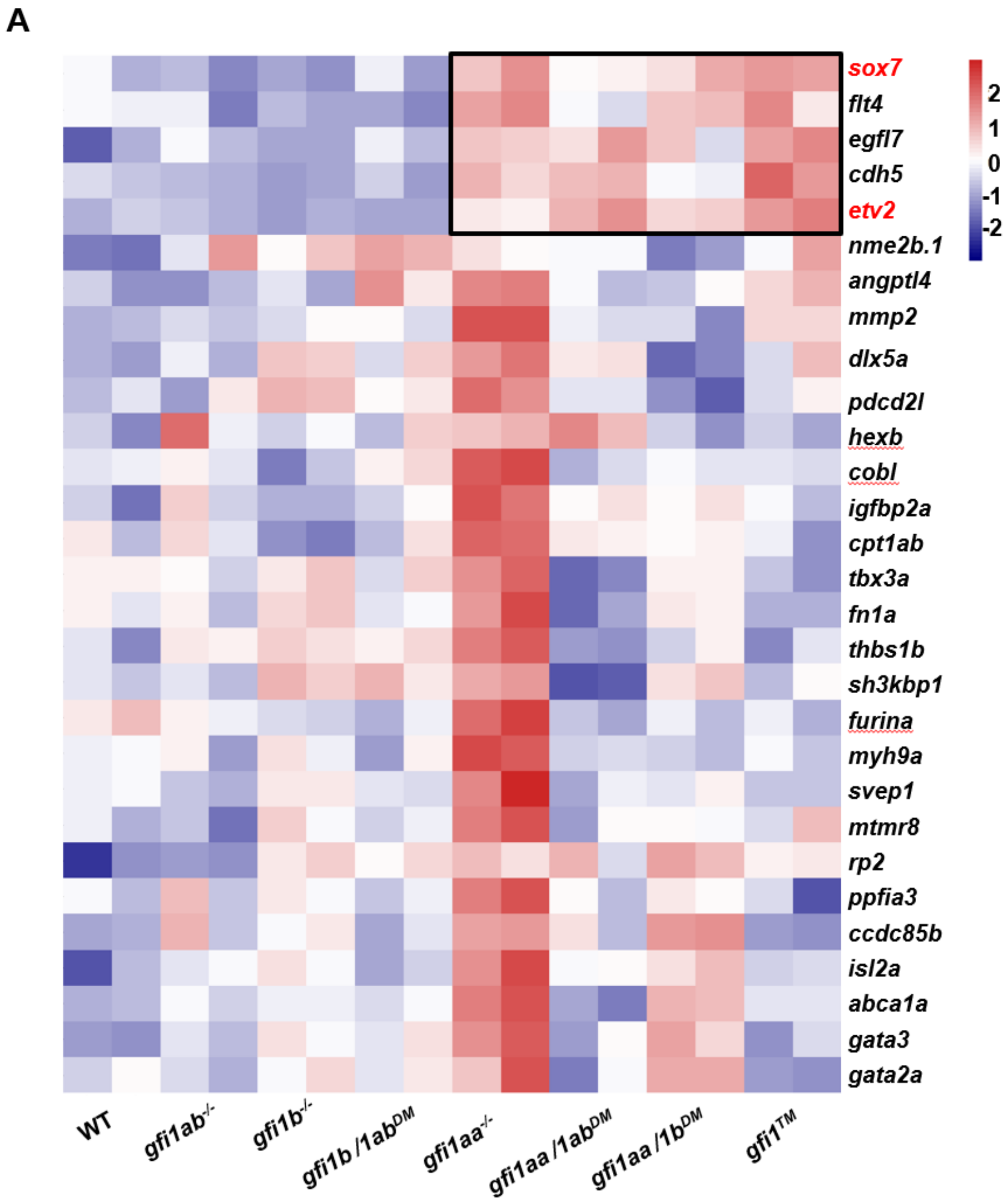

Supplementary Figure 4. The RNA expression of vasculature markers in Figure 2D among all *gfi1* mutants. (A) Heat map of *gfi1* mutants showed the vascular development genes expression level. The color scale indicated the expression level.

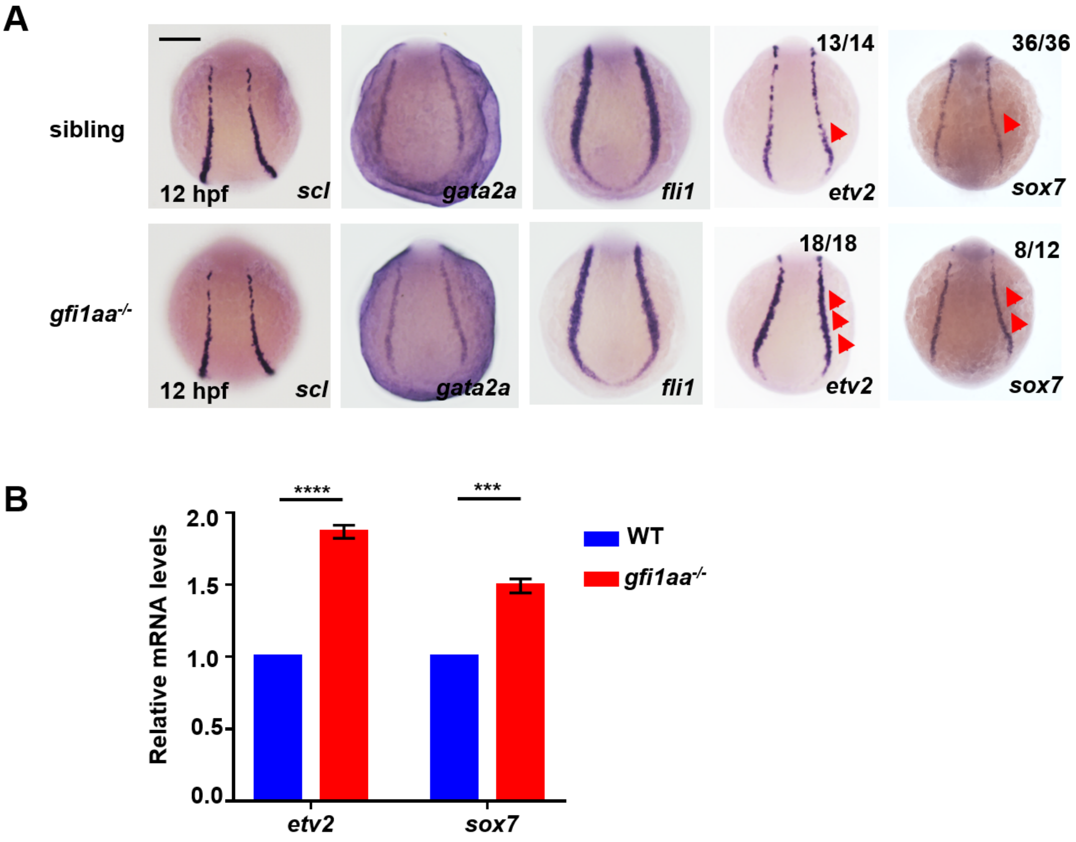

Supplementary Figure 5. Hemangioblast markers *etv2* and *sox7* were increased in *gfi1aa* mutant. (A-B) Expression of *etv2* and *sox7* increased in *gfi1aa*$^{-/-}$ mutant at the early stage of primitive hematopoiesis. **(A)** Expression of *scl*, *gata2*, *fli1*, *etv2*, and *sox7* in sibling and *gfi1aa*$^{-/-}$ mutant at 12 hpf by WISH. The numbers in the top right corner indicate representative expression embryo numbers of the indicated marker.Scale bar: 200 µm. **(B)** Expression of *etv2* and *sox7* increased in *gfi1aa*$^{-/-}$ mutant compared to AB WT at 12 hpf by qPCR. (The error bars indicated the mean±SEM of three independent experiments, ****P<0.0001, ***P<0.001, t-test).

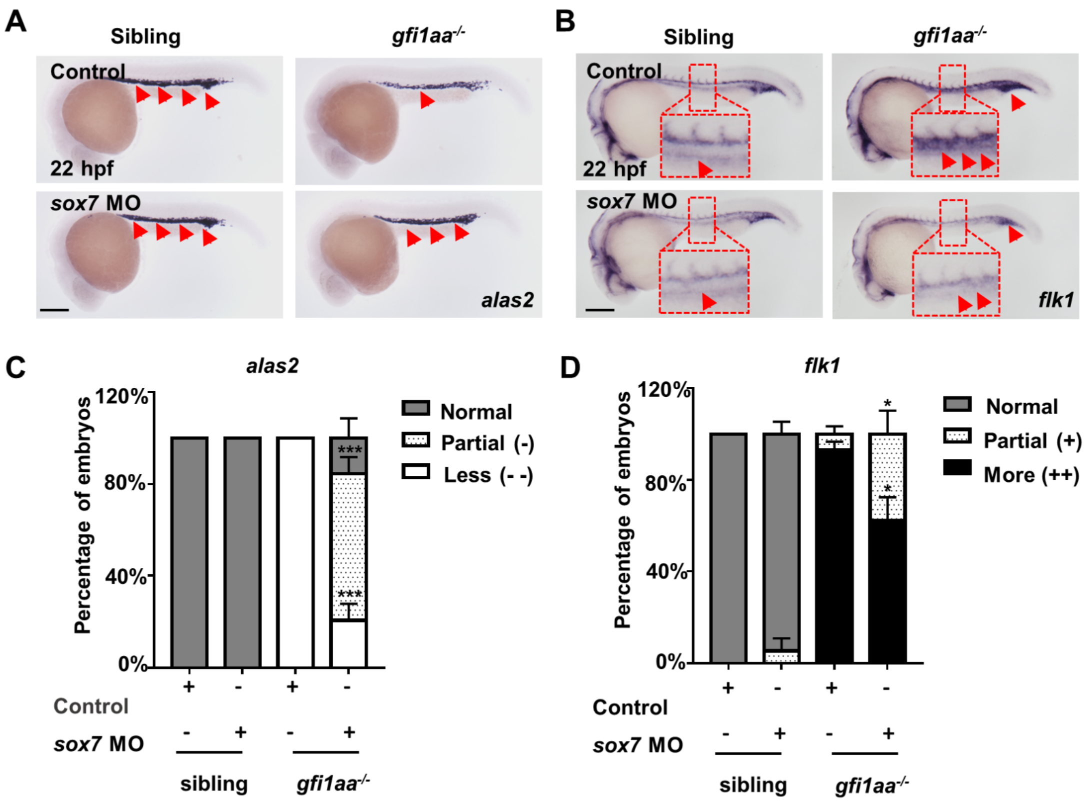

Supplementary Figure 6. Knockdown *sox7* could rescue the hematopoietic defect of *gfi1aa* mutant. (A, B) Expression of *alas2* **(A)** and *flk1* **(B)** in siblings and *gfi1aa*$^{-/-}$ mutants injected with 0.5 pmol *sox7* MO or control. The red arrow indicated WISH signals and the red box indicated the magnification of ICM region. Scale bar: 200 µm. **(C,D)** Analysis of *alas2* **(C)** and *flk1* **(D)** expression in siblings and *gfi1aa*$^{-/-}$ mutants rescued by *sox7* MO. The asterisks indicate the statistical difference between the rescued proportion by *sox7* MO; *gfi1aa*$^{-/-}$ and *gfi1aa*$^{-/-}$. (Three independent experiments were performed, ***P<0.001, *P<0.05, *t*-test, n≥10 embryos for each group).

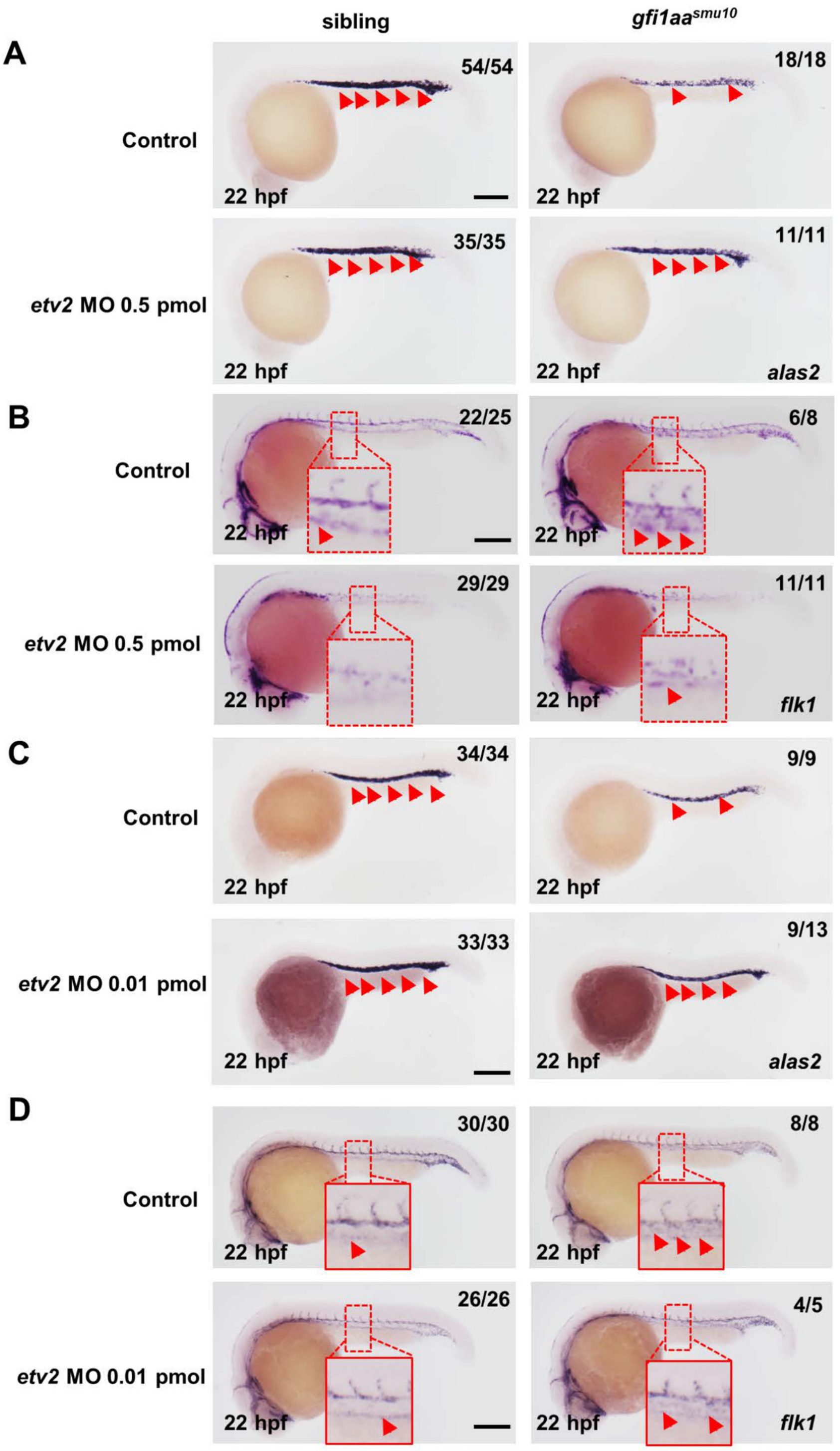

Supplementary Figure 7. The high dosage *etv2* MO displayed severe vascular development defects. (A, B) Expression of *alas2* **(A)** and *flk1* **(B)** in siblings and *gfi1aa*$^{-/-}$ mutants injected with 0.5 pmol *etv2* MO or control. **(C, D)** Expression of *alas2* **(C)** and *flk1* **(D)** in siblings and *gfi1aa*$^{-/-}$ mutants injected with 0.01 pmol *etv2* MO or control. The red arrow indicated WISH signals and the red box indicated the magnification of ICM region. The numbers in the top right corner indicate representative expression embryo numbers of the indicated marker. Scale bar: 200 μm.

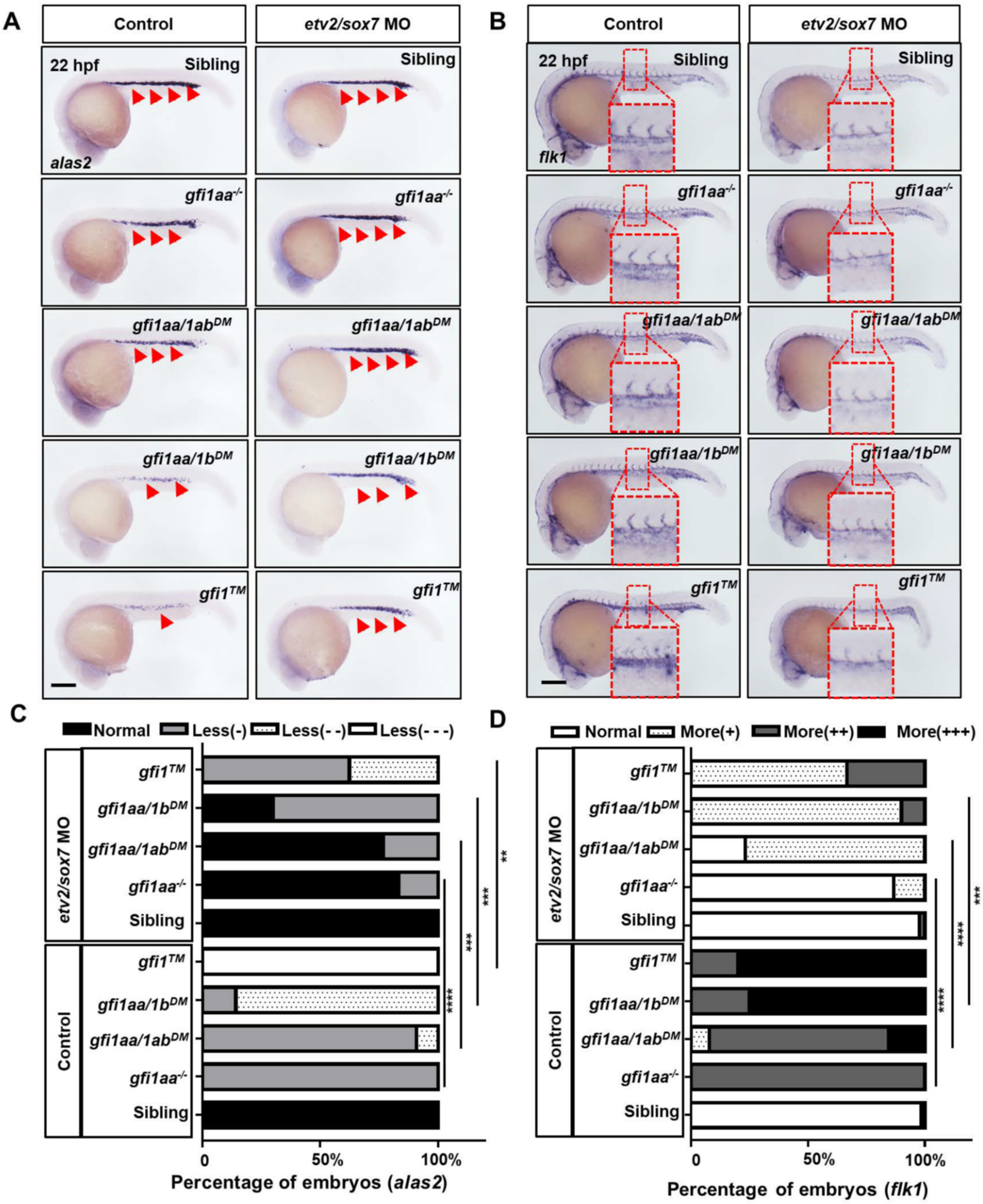

Supplementary Figure 8. *etv2/sox7* knockdown could partially rescue the hematopoietic defect of *gfi1^{TM}* mutant. (A, B) Expression of *alas2* **(A)** and *flk1* **(B)** in siblings, *gfi1aa$^{-/-}$*, *gfi1aa/1ab^{DM}*, *gfi1aa/1b^{DM}*, *gfi1^{TM}* mutant injected with 0.5 pmol *sox7* MO with 0.005 pmol *etv2* MO or control. The red arrow indicated WISH signals and the red box indicated the magnification of ICM region. Scale bar: 200 µm. **(C,D)** Analysis of *alas2* **(C)** and *flk1* **(D)** expression in siblings, *gfi1aa$^{-/-}$*, *gfi1aa/1ab^{DM}*, *gfi1aa/1b^{DM}*, *gfi1^{TM}* mutant rescued by *etv2/sox7* MO. The asterisks indicate the statistical difference between the rescued proportion by *etv2/sox7* MO. (****P<0.0001, ***P<0.001, **P<0.01, *P<0.05, Fisher's exact test, n≥6 embryos for each group).
